# Increasing Levels of Physical Disturbance Affect Soil Nematode Community Composition in a Previously Undisturbed Ecosystem

**DOI:** 10.2478/jofnem-2022-0022

**Published:** 2022-07-20

**Authors:** Satyendra Kumar Pothula, Gary Phillips, Ernest C. Bernard

**Affiliations:** 1 Department of Entomology and Plant Pathology, University of Tennessee, Knoxville, TN 37996-4560 Tennessee

**Keywords:** abundance, c-p class, ecology, ecosystem, litter, physical disturbance, richness, tillage, trophic group

## Abstract

Soil is essential for the sustenance of life. Diverse soil organisms support several biological processes such as organic matter decomposition, mineralization, nutrient cycling, and controlling pests and diseases. Among multicellular soil organisms, nematodes are ubiquitous, functionally diverse, and abundant. Notably, agricultural practices have diverse impacts on plants, soils, and soil organisms. Tillage affects nematodes directly by altering pore size and disrupting the continuity of water films and indirectly by affecting the lower trophic groups such as bacteria and fungi. The primary goal of this study was to examine the effect of increasing levels of physical disturbance on nematode communities in an undisturbed forest ecosystem. The experiment included four treatments: control with no disturbance, surface litter removed with no litter and no vegetation, tilling the soil with a rototiller every 2 mon, and every 2 wk. Tillage significantly reduced the overall abundance and overall richness of nematode communities over time. Among nematode trophic groups, tillage significantly reduced the abundance and richness of bacterial feeders, predators, and omnivores over time. The abundance and richness of c-p 2, c-p 4, and c-p 5 class nematodes were significantly decreased by tillage. Unlike tillage, minimal disturbance such as removal of surface litter resulted in a significant decrease in the abundance of only three genera: *Acrobeles*, *Aporcelaimellus*, and *Boleodorus*. Nonmetric multidimensional scaling analysis revealed that nematodes of higher c-p classes such as Dorylaimida, *Aporcelaimellus*, *Alaimus, Clarkus*, and *Tripyla* were sensitive to physical disturbances. Bacterial feeders belonging to the c-p 2 class such as *Tylocephalus*, *Acrobeles*, *Ceratoplectus, Plectus, and Pseudacrobeles* were significantly reduced by tillage. Moreover, tillage significantly reduced the functional metabolic footprint of nematodes, which indicates decreased metabolic activity, reduced C inflow, and poorly structured soil food webs. Previous studies conducted in agricultural ecosystems determined that *Clarkus*, *Filenchus*, and *Plectus* were tolerant to tillage; however, they were found sensitive to tillage in our study. Overall, our study suggests that increasing levels of physical disturbance are detrimental to nematode community abundance and diversity that could affect soil ecosystem stability and sustainability.

Soil is indispensable for the sustenance of life. Soil provides essential resources for human activities such as agriculture, buildings, and industries (Brussaard, 1997). Several biological processes are continuously active in the soil and play an important role in the replenishment of soil resources and ecosystem maintenance (Young and Crawford, 2004). Biological processes in the soil are due to the dynamic interactions of diverse assemblages of living organisms including unicellular bacteria and protozoa to multicellular nematodes, earthworms, and arthropods (Giller *et al*., 1997; Bach *et al*., 2020). Diverse soil organisms support several biological processes such as organic matter decomposition, mineralization, nutrient cycling, and controlling pests and diseases (Brussaard, 1997; Heinen *et al*., 2018), which directly and indirectly affect crop growth and quality (Swift *et al*., 2004; Giller *et al*., 2005). Among multicellular soil organisms, nematodes are by far the most abundant (Porazinska *et al*., 2009; Lu *et al*., 2020; van den Hoogen *et al*., 2020). Nematodes are at the center of the soil food web by interacting with several other soil trophic groups in the lower hierarchy of the soil food web, with plants, bacteria, and fungi serving as food for nematodes; in turn, trophic groups in the higher hierarchy of the soil food web, such as predatory mites, eat nematodes (Moore, 1994; Roger-Estrade *et al*., 2010).

Nematodes play a pivotal role in organic matter decomposition (Freckman, 1988; Beare *et al*., 1992; Yeates and Coleman, 2021), mineralization (Yeates, 1979; Griffiths, 1989; Neher, 2001; Sikder and Vestergård, 2020), and uptake of nutrients by plants (Ingham *et al*., 1985; Gebremikael *et al*., 2016). Nematodes feeding on bacteria and fungi promote mineralization and release nutrients into the soil, thereby regulating decomposition (Ingham *et al*., 1985). Since nematodes are ubiquitous, functionally diverse, and abundant, they can be used to gauge the condition of structure and function of soil food webs (Bongers, 1990; Bongers and Bongers, 1998; Ferris *et al*., 2001; Neher, 2001). Nematodes have been categorized into different trophic groups such as bacterial feeders, fungal feeders, plant feeders, predators, and omnivores based on their stoma and esophagus morphology (Yeates *et al*., 1993). Additionally, a colonizer-persister (c-p) scale comprising five levels has been developed for nematodes ranging from colonizers (c-p 1) with high fecundity rate, short generation time, and toleration of disturbances, to persisters (c-p 5) with low fecundity rate, long generation time, and sensitivity to disturbances (Bongers, 1990). The c-p scale reflects the continuum of r- and K-strategists. Nematode community indices have been used to monitor ecological conditions of soil and the influence of agricultural activities on nematodes (Sohlenius *et al*., 1987; Bongers, 1990; Freckman and Ettema, 1993; Neher *et al*., 1995; Wardle *et al*., 1995).

Agricultural activities affect soil structure, biological activity, and processes such as decomposition, mineralization, and nutrient cycling by altering the physicochemical properties of soil (Stinner *et al*., 1984; Dick *et al*., 1988; Fraser *et al*., 1994). Notably, agricultural practices such as cultivation, crop rotation, tillage, and pesticide application have diverse impacts on plants, soils, and soil organisms (Elliott and Cole, 1989). Tillage alters soil properties such as moisture, temperature, aeration, and organic matter content and ultimately affects organisms that are living in the soil (Kladivko, 2001; Holland, 2004; Golabi *et al*., 2014). Furthermore, tillage disrupts the relationship between soil organisms by either killing or injuring them or exposing them to predators (Altieri, 1999; Roger-Estrade *et al*., 2010). Nematodes are more responsive to mechanical disturbance of soil than surface-dwelling invertebrates (Wardle *et al*., 1995). Tillage affects nematodes directly by altering pore size and disrupting the continuity of water films needed by nematodes and indirectly by affecting the lower trophic groups such as bacteria and fungi (Wardle *et al*., 1995).

Surface litter is essential for energy flow in the soil food webs (Attiwill and Adams, 1993). Removal of surface litter affects the dynamics of decomposition, which has a significant effect on the soil C pools (Wu *et al*., 2018). Surface litter removal decreases the resources available for nematodes, which affects the processes in the soil and alters the soil C distribution (Wu *et al*., 2021).

The effect of different types of physical disturbances on nematode communities has been previously investigated in agricultural ecosystems, i.e., those previously tilled or disturbed (Lenz and Eisenbeis, 2000; Okada and Harada, 2007; Rahman *et al*., 2007; Dong *et al*., 2013; Forge *et al*., 2015; Sánchez-Moreno *et al*., 2015; Zhang *et al*., 2015, 2019; Zhong *et al*., 2017; Wu *et al*., 2021). However, the effect of physical disturbances on soil organisms can be better evaluated by conducting experiments in undisturbed ecosystems, where soil organisms were never exposed to any kind of disturbances. Therefore, the main objective of this study was to examine the effect of increasing levels of physical disturbance on nematode communities in an undisturbed forest ecosystem. We hypothesized that the increase in the level of physical disturbance would negatively affect nematode communities.

## Materials and Methods

### Site description

A field experiment was conducted from April 2017 to May 2018 in a secondary mixed deciduous forest ecosystem dominated by nut-bearing oak and hickory species of trees in Farragut, TN, USA (35 º 54¢3^2^N, 84 º 11¢37^2^W; 311 m elevation). The experimental site is located in a temperate and seasonal climate with a mean annual temperature of 15.3°C and a mean annual precipitation of 1,224 mm. The soil at this site is classified as Minvale-Bodine-Fullerton complex. The experimental site had not been disturbed for at least 50 yr before the experiment was laid out. An understory was absent, and groundcover was negligible. The site sloped slightly toward the northwest.

### Experimental design

The experiment included four treatments with increasing levels of physical disturbance. The first treatment was a control with no disturbance; the second treatment was surface litter removed (SLR) with no litter and no vegetation; the third treatment was tilling 15 cm deep with a rototiller every 2 mon after surface litter removal (R2M); the fourth treatment was tilling 15 cm deep every 2 wk after surface litter removal (R2W). Litter and vegetation were cleared every 2 wk from all the treatments except for the control. Each treatment was replicated thrice. Each plot was 2 m ´ 2 m and separated by a 2-m distance. The design of the experiment was a completely randomized design with repeated measures. The experiment was started in April 2017 and concluded in May 2018.

### Soil sampling

Soil samples were collected from all the plots in April 2017 before starting the experiment and subsequently samples were collected every 2 mon: June 2017, September 2017, November 2017, January 2018, and May 2018. The interval between the tillage and sampling was 2 mon for R2M treatment and 2 wk for R2W treatment. At each sampling time, five soil cores, each having a 2-cm diameter and a 20-cm depth, were randomly collected from each plot. Soil samples from each plot were pooled into a plastic bag to prevent drying of soil, and then transported in a cooler to the laboratory where they were subsequently stored at 4°C before extracting nematodes on the next day.

Nematode extraction and identification were carried out as follows: Composite soil samples were thoroughly homogenized and 100 cm^3^ of each soil sample was used for extraction of nematodes through a sugar flotation-centrifugation method (Jenkins, 1964). Extracted nematodes from each sample were counted and the first 150 nematodes were identified to genus level using a differential interference contrast microscope. Proportions of each taxon were extrapolated to the entire sample. The identified nematode genera were assigned to their respective trophic groups: bacterial feeders (BF), fungal feeders (FF), plant feeders (PF), omnivores (OM), and predators (PR) (Yeates *et al*., 1993), and colonizer-persister (c-p) scale was established based on their life history characteristics and survival strategies associated with r- and K-selection. Nematodes with c-p 1 (enrichment opportunistic nematodes) and c-p 2 (mostly microbial and plant feeders) values are considered colonizers (r-selected), with small size, short life span, high fecundity, and high tolerance to environmental disturbances. Nematodes with c-p value 5 (mostly predators and omnivores) are persisters (K-selected), long-lived nematodes with low fecundity, slow development, and high sensitivity to environmental disturbances. (Bongers 1990).

### Nematode ecological indices

The following ecological indices were calculated to assess the structure and functional role of nematode communities in soil food webs of increasing levels of physical disturbance: Simpson’s dominance index (λ), λ = ΣP^2^ (Simpson, 1949); Shannon–Weaver index (H´), H´ = –Σ^i^P_i_lnP_i_, where P is the proportion of individuals in the i^th^ taxon (Shannon, 1948); maturity index (MI) for free-living taxa were computed as MI = Σ[CP-value (i) × f(i)]/[total numbers of nematodes], where i is the individual taxon and fi is the frequency of taxa in the sample (Bongers, 1990). MI is used to evaluate the functioning and condition of a soil ecosystem as a consequence of environmental disturbance. MI values range from 1 to 5. A high MI suggests more abundant and diverse nematodes of higher c-p classes and a less disturbed ecosystem. A low MI suggests more abundant and diverse nematodes of lower c-p classes and a highly disturbed ecosystem. Plant-parasitic index (PPI) was calculated for plant-parasitic genera (Yeates and Bongers, 1999). Nematode channel ratio (NCR) indicates the decomposition pathway of the soil food web (Yeates and Bongers 1999). NCR is calculated as NCR = bacterial feeders/(bacterial feeders + fungal feeders) and ranges from 0 (fungi-dominated) to 1 (bacteria-dominated). Soil food web indices were calculated based on nematode functional guilds determined by the combination of c-p groups and trophic groups (Ferris *et al*., 2001). Soil food web indices include the Basal Index (BI), an indicator of the disturbed condition of soil food webs; Channel Index (CI), an indicator of decomposition of organic matter mediated by fungi; Enrichment Index (EI), an indicator of the predominance of bacterial feeders and enrichment conditions; and Structure Index (SI), an indicator of structured soil food webs with high trophic linkage (Ferris *et al*., 2001). Soil food web indices were calculated using the Nematode Joint Indicator Analysis tool (Sieriebriennikov *et al*., 2014).

### Nematode metabolic footprints

Functional metabolic footprints (FMF) of nematode communities in soil food webs of increasing levels of physical disturbance were calculated using the Nematode Joint Indicator Analysis tool (Sieriebriennikov *et al*., 2014). FMF was calculated to evaluate the changes in the metabolic activity and flow of C into the soil food webs (Ferris, 2010). The total area of FMF is partitioned into enrichment footprint (efoot) indicating lower trophic groups (c-p 1–2) and structure footprint (sfoot) indicating higher trophic groups (c-p 3–5). The efoot is the metabolic footprint of lower trophic-group nematodes (c-p 1–2) whose population rapidly increases due to the increase in resources. The sfoot is the metabolic footprint of higher trophic group nematodes (c-p 3–5) with regulatory function. In an FMF graph, the y-axis represents the efoot, and the x-axis represents the structural footprint. In the FMF graph, the y-axis coordinates (EI– 0.5Fe/k and EI+ 0.5Fe/k) and x-axis coordinates (SI– 0.5Fs/k and SI+ 0.5Fs/k) were sequentially joined to depict the metabolic footprints of nematode communities. Fs indicates higher trophic groups (c-p 3–5) and Fe indicates lower trophic groups (c-p 1–2). The adjusted k value is 4 (Ferris, 2010).

### Statistical analysis

Overall richness and abundance of nematodes were estimated for each sample. In addition, nematode richness and abundance for each trophic group and each c-p class at each time point were estimated. Statistical analyses were performed to compare the overall nematode richness and abundance as well as the richness and abundance of each trophic group and each c-p class across different treatments at different time points. Normality of residuals and equal variance were assessed using the Shapiro–Wilk statistic and visual observation of histograms and data were ln(x + 1)-transformed prior to statistical analysis. Analysis of variance with repeated measures was conducted with SAS (Glimmix procedure, SAS Institute, Cary, NC) and least square means were compared with Tukey’s LSD with a significance level of *P* < 0.05.

Changes in community structure with increasing levels of physical disturbance over time were visualized by nonmetric multidimensional scaling (NMDS) ordination with the Bray–Curtis distance matrix. Permutational multivariate analysis of variance (PERMANOVA; Anderson, 2001) was used to assess the significance of the differences among nematode community composition of the four treatments. The similarity percentage analysis (SIMPER) was used to determine the contribution of nematode genera to dissimilarities between treatments with a significance level of *P* < 0.05. All analyses were performed using the functions metaMDS, adonis, and simper in the vegan package of R, version 3.3.3 (Oksanen *et al*., 2020).

## Results

### Nematode abundance and community composition

In total, 56 genera were identified at different levels of physical disturbance across different sampling times. Of the 56 genera, 26 most abundant genera are listed in Supplementary Table 1. The nematode genera with zero abundance in most of the treatments at different sampling times were not considered for individual nematode analysis. Rhabditidae*, Meloidogyne, Plectus, Filenchus, Aphelenchoides, Acrobeloides, Pseudacrobeles*, and *Gracilacus* were the dominant taxa for all treatments at all sampling times.

**Figure 1:**
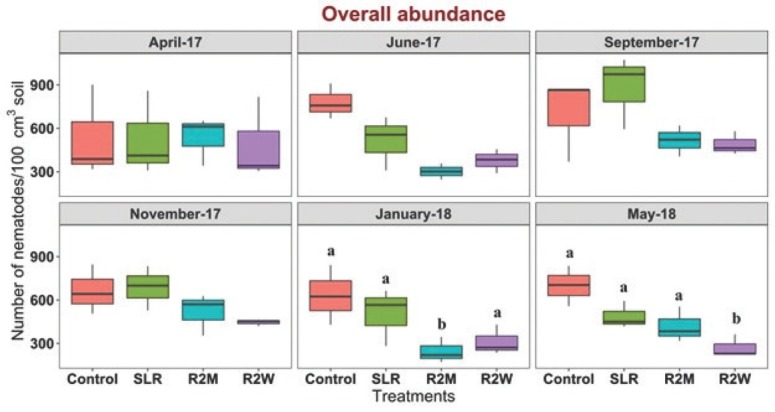
Effect of increasing levels of physical disturbance on genus-level nematode abundance. Box plots representing the number of nematodes per 100 cm^3^ of soil in control, SLR, R2M, and R2W at each sampling time. Lower and upper box boundaries represent 25th and 75th percentiles, respectively; line inside the box indicates median; and lower and upper error lines represent 10th and 90th percentiles, respectively. Letters indicate significant differences among treatments at each sampling time at *P <* 0.05 (Tukey–LSD test). R2M, rototill for every 2 mon; R2W, rototill for every 2 wk; SLR, surface litter removed.

**Figure 2:**
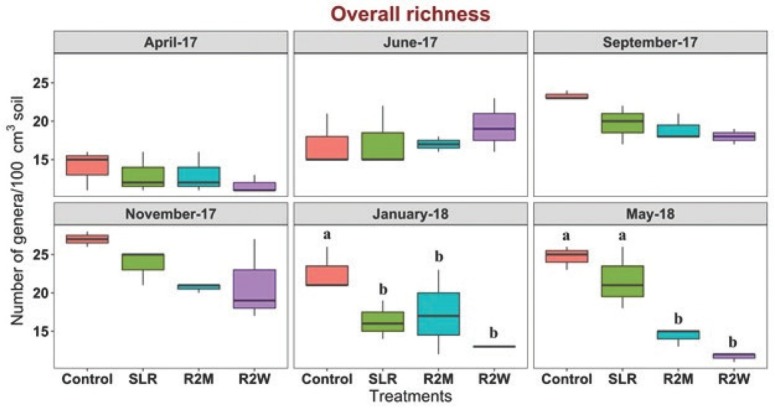
Effect of increasing levels of physical disturbance on genus-level nematode richness. Box plots representing the number of genera per 100 cm^3^ of soil in control, SLR, R2M, and R2W at each sampling time. Lower and upper box boundaries represent 25th and 75th percentiles, respectively; line inside the box indicates median; and lower and upper error lines represent 10th and 90th percentiles, respectively. Letters indicate significant differences among treatments at each sampling time at *P <* 0.05 (Tukey–LSD test). R2M, rototill for every 2 mon; R2W, rototill for every 2 wk; SLR, surface litter removed.

The effect of increasing levels of physical disturbance on nematode abundance was statistically significant during January 2018 and May 2018 (*P* < 0.05) (Fig. 1). The overall abundance of nematodes was significantly lower in R2M compared to the control, SLR, and R2W treatments in January 2018 (*P* < 0.05) (Fig. 1). In addition, the overall abundance of nematodes was significantly lower in R2W compared to the control, SLR, and R2M treatments in May 2018 (*P* < 0.05) (Fig. 1). Although the effect of tillage on nematode overall abundance was not statistically significant, nematode overall abundance was consistently lower in R2M and R2W compared to the control and SLR treatment since June 2017 (Fig. 1). Similarly, the effect of increasing levels of physical disturbance on nematode richness was statistically significant during January 2018 and May 2018 (*P* < 0.05) (Fig. 2). Overall richness of nematodes was significantly lower in SLR, R2M, and R2W compared to the control in January 2018 (*P* < 0.05) (Fig. 2). The effect of tillage on nematode richness was more pronounced in the last sampling in May 2018 in which nematode richness was significantly decreased in R2M and R2W compared to the control and SLR treatments (*P* < 0.05) (Fig. 2). Although the effect of tillage on nematode overall richness was not statistically significant, the overall richness of nematodes was consistently lower in R2M and R2W compared to the control and SLR treatments from November 2017 (Fig. 2)

Among 56 genera, 20 taxa were bacterial feeders, 18 taxa were plant feeders, 7 taxa were fungal feeders, 6 genera were omnivores, and 5 taxa were predators. The effect of increasing levels of physical disturbance on nematode abundance and richness of each trophic group was analyzed. Increasing levels of physical disturbance significantly affected the abundance of bacterial feeders, fungal feeders, predators, and omnivores during the last two samplings (January 2018 and May 2018) (*P* < 0.05). However, the abundance of plant feeders was not significantly affected Supplementary Table 2). Tillage significantly lowered the abundance of bacterial feeders, predators, and omnivores in R2M and R2W treatments compared to control during the last sampling in May 2018 (*P* < 0.05) (Supplementary Table 2). In addition, the abundance of fungal feeders was also significantly affected by the tillage treatments, R2M and R2W during the last two samplings, January 2018 and May 2018, respectively (*P* < 0.05) (Supplementary Table 2). On the other hand, tillage significantly affected the richness of bacterial feeders, predators, and omnivores, especially during the last two samplings (January 2018 and May 2018) (*P* < 0.05) (Supplementary Table 2). The richness of bacterial feeders was significantly lower in R2W compared to the control and SLR treatments during the last two samplings (*P* < 0.05). Additionally, the richness of predators was significantly lower in R2M and R2W compared to SLR treatment, whereas the richness of omnivores was significantly decreased in R2M and R2W compared to the control and SLR treatments (*P* < 0.05) (Supplementary Table 2).

Increasing levels of physical disturbance significantly affected the abundance of 10 genera, *Acrobeles*, *Alaimus*, *Aporcelaimellus*, *Boleodorus*, *Ceratoplectus*, *Clarkus*, Dorylaimida, *Filenchus, Prismatolaimus*, and *Tripyla* during the last two samplings of January 2018 and May 2018 (*P* < 0.05) (Supplementary Table 1). The abundance of *Acrobeles*, *Aporcelaimellus*, and *Boleodorus* was significantly reduced with the increase in the level of physical disturbance in May 2018 (*P* < 0.05) (Supplementary Table 1). Tillage significantly reduced the abundance of *Alaimus* in R2M and R2W compared to the control and SLR treatments during the last sampling of May 2018 (*P* < 0.05) (Supplementary Table 1). The abundance of *Ceratoplectus* and *Filenchus* was significantly lower in the tillage treatments, R2M and R2W compared to the control in January 2018 and May 2018 (*P* < 0.05) (Supplementary Table 1). The abundance of *Clarkus*, Dorylaimida, and *Tripyla* was significantly decreased in R2M and R2W treatments compared to the control during the last sampling, May 2018 (*P* < 0.05) (Supplementary Table 1). Tillage significantly reduced the abundance of *Prismatolaimus* in the R2W treatment compared to the control during January 2018 and May 2018 (*P* < 0.05) (Supplementary Table 1). Removal of surface litter resulted in a significant decrease in the abundance of *Acrobeles*, *Aporcelaimellus*, and *Boleodorus* compared to the control during the last sampling period, May 2018 (*P* < 0.05) (Supplementary Table 1).

The effect of increasing levels of physical disturbance on nematode abundance and richness of each c-p class was also analyzed. Increasing levels of physical disturbance did not affect the abundance of c-p 1 and c-p 3 class nematodes whereas the impact was significant in the cases of c-p 2, c-p 4, and c-p 5 classes (*P* < 0.05). The nematode abundance of the c-p 2 class was lower in R2M and R2W compared to the control in the last two samplings of January 2018 and May 2018 (*P* < 0.05) (Supplementary Table 3). Similarly, the abundance of c-p 5 class nematodes was lower in R2M and R2W than in the control during the last sampling, May 2018 (*P* < 0.05) (Supplementary Table 3). The abundance of c-p 4 class nematodes was decreased in R2M and R2W compared to the control and SLR treatments during May 2018 (*P* < 0.05) (Supplementary Table 3). Increasing levels of physical disturbance did not affect the richness of c-p 1, c-p 3, and c-p 5 class nematodes whereas they significantly affected the richness of c-p 2 and c-p 4 classes (*P* < 0.05) (Supplementary Table 3). The richness of nematodes in the c-p 2 class was significantly lower in R2M and R2W compared to the control during January 2018 and May 2018 (*P* < 0.05). Moreover, the richness of nematodes in the c-p 4 class was significantly reduced in R2W compared to the control in January 2018 (*P* < 0.05). In the last sampling, tillage significantly reduced the richness of c-p 4 class nematodes in R2M and R2W compared to the control and SLR (*P* < 0.05) (Supplementary Table 3).

### Nematode ecological indices

A significant effect of increasing levels of physical disturbance was observed on the values of λ, H´, EI, and SI (*P* < 0.05) (Supplementary Table 4). The value of λ significantly increased with the increasing levels of physical disturbance. The value of λ was significantly lower in the control than in R2W in January 2018 and lower in the control compared to R2M and R2W during May 2018 (*P* < 0.05) (Supplementary Table 4). The value of H´ significantly decreased with the increasing levels of physical disturbance. The value of H´ was significantly lower in R2W compared to the control during January 2018, and lower in R2M and R2W than in the control during the last sampling, May 2018 (*P* < 0.05) (Supplementary Table 4). MI value significantly reduced with the increase in the level of physical disturbance. MI was significantly lower in R2W than in control treatment during January 2018, and lower in R2M and R2W compared to the control and SLR during May 2018 (*P* < 0.05) (Supplementary Table 4). PPI value was significantly higher in R2M and R2W compared to the control during the last sampling, May 2018 (*P* < 0.05) (Supplementary Table 4). EI value significantly increased with the increasing levels of physical disturbance. EI was significantly higher in R2W compared to the control during the last two samplings, January and May 2018 (*P* < 0.05) (Supplementary Table 4). In contrast, SI significantly decreased with the increasing levels of physical disturbance. SI was significantly lower in R2W compared to the control and SLR during January 2018, and lower in R2M and R2W than in the control and SLR during the last sampling, May 2018 (*P* < 0.05) (Supplementary Table 4).

### Nematode metabolic footprints

**Figure 3:**
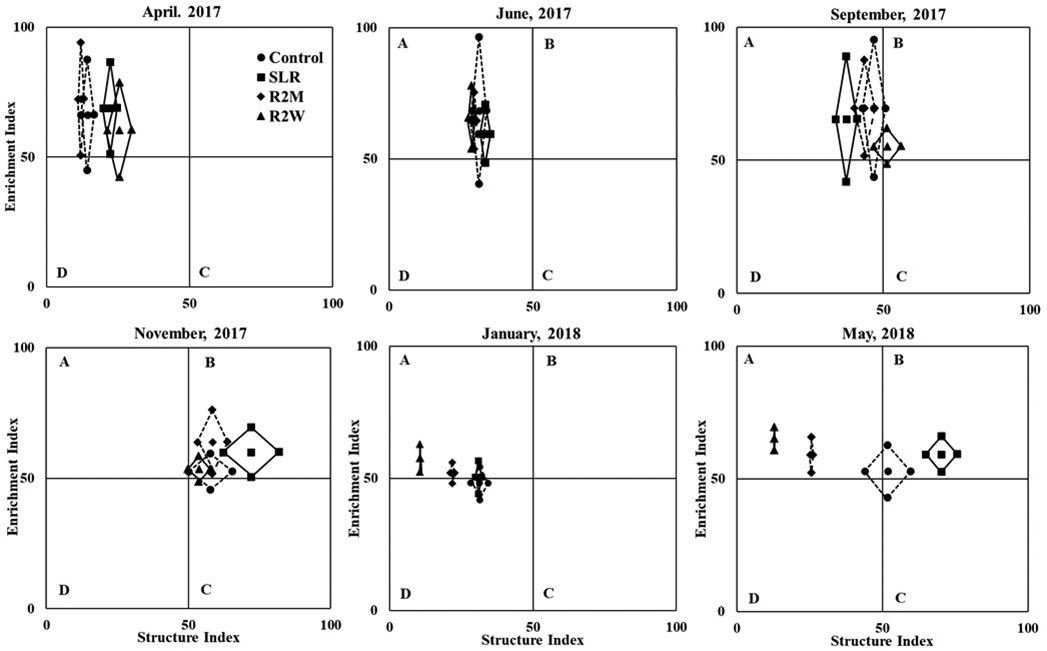
FMF of nematode communities subjected to different levels of physical disturbance: control, SLR, R2M, and R2W. The vertical axis represents the efoot, and the horizontal axis represents the sfoot. The FMF is depicted by sequentially joining points: (SI– 0.5Fs/k, EI); (SI+ 0.5Fs/k, EI); (SI, EI– 0.5Fe/k); and (SI, EI+ 0.5Fe/k). Fs represents sfoot and Fe represents efoot (Ferris, 2010). The adjusted k value is 4. FMF, functional metabolic footprints; R2M, rototill for every 2 mon; R2W, rototill for every 2 wk; sfoot, structure footprint; SLR, surface litter removed.

A significant effect of increasing levels of physical disturbance was observed on efoot and sfoot (*P* < 0.05) (Supplementary Table 4). Tilling every 2 wk resulted in a significant reduction of efoot compared to the control and other treatments during May 2018 (*P* < 0.05) (Supplementary Table 4). The sfoot was significantly lower in R2W than in the control and SLR treatments during January 2018 and significantly lower in R2M and R2W treatments compared to the control and SLR treatments during the last sampling in May 2018 (*P* < 0.05) (Supplementary Table 4). The FMF area of nematode communities was decreased with the increasing levels of physical disturbance (Fig. 3). The area of FMF of R2M and R2W was decreased over time compared to the control and SLR treatments. All the treatments were clustered together in the same quadrat until November 2017 and started spreading out in January 2018. In May 2018, control and SLR treatments were located in quadrat B, which indicated a maturing ecosystem with enriched soil nutrients and a well-structured soil food web, while R2M and R2W were located in quadrat A, which indicated a disturbed and poorly structured soil food web. The FMF indicates the total area of the enrichment and sfoot as demonstrated in Figure 3.

### The relationship between nematode abundance and treatments

**Figure 4:**
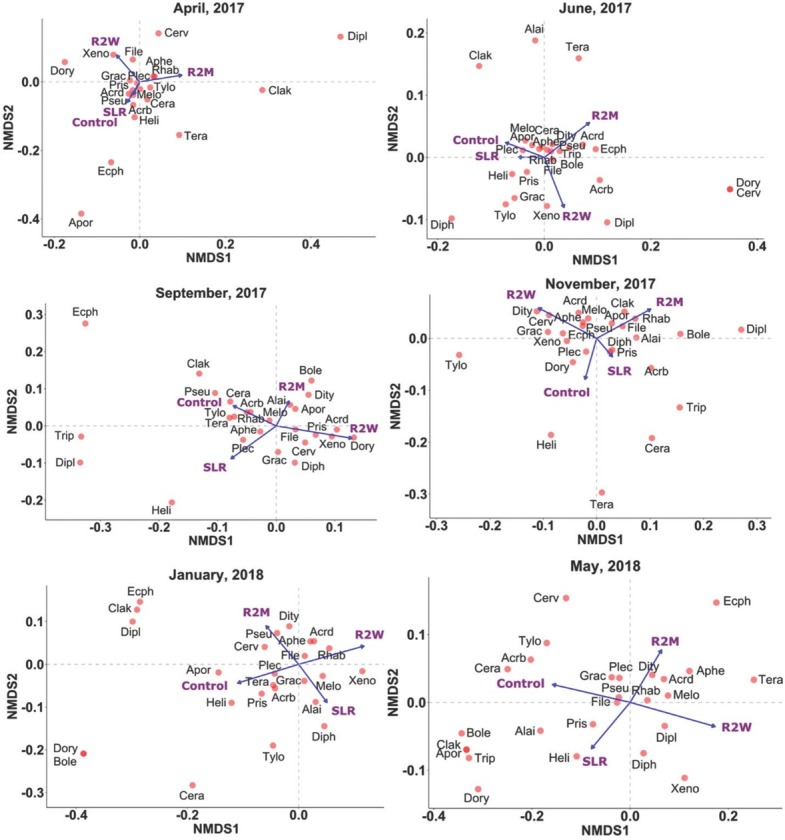
Biplot representing the NMDS performed on nematodes communities subjected to different levels of physical disturbance: control, SLR, R2M, and R2W in April 2017 (PERMANOVA: *P* = 0.92, NMDS; Stress = 0.05), June 2017 (PERMANOVA: *P* = 0.117, NMDS; Stress = 0.09), September 2017 (PERMANOVA: *P* = 0.075, NMDS; Stress = 0.11), November 2017 (PERMANOVA: *P* = 0.226, NMDS; Stress = 0.11), January 2018 (PERMANOVA: *P* = 0.044, NMDS; Stress = 0.08), and May 2018 (PERMANOVA: *P* = 0.015, NMDS; Stress = 0.07). Treatments are depicted using arrows while nematodes are depicted with dots. NMDS, Nonmetric multidimensional scaling; PERMANOVA, Permutational multivariate analysis of variance; R2M, rototill for every 2 mon; R2W, rototill for every 2 wk; SLR, surface litter removed.

NMDS analysis of nematode communities showed a significant differentiation in nematode communities with increasing levels of physical disturbance during January 2018 (Fig. 4; PERMANOVA: *R*^2^ = 0.42, *F* = 1.93, *P* = 0.044, Stress = 0.08), and May 2018 (Fig. 4; PERMANOVA: *R*^2^ = 0.46, *F* = 2.32, *P* = 0.015, Stress = 0.07). During January 2018, R2M and R2W treatments negatively impacted *Boleodorus*, Dorylaimida, *Acrobeles*, *Prismatolaimus*, *Ceratoplectus*, *Alaimus, Plectus*, *Gracilacus*, *Tylocephalus*, and *Helicotylenchus*. Conversely, *Aphelenchoides*, Rhabditidae, Diplogasteridae, *Ditylenchus*, *Acrobeloides*, and *Ecphyadophora* were positively associated with R2M and R2W (Fig. 4). Along with the aforementioned taxa, tillage negatively impacted *Aporcelaimellus*, *Clarkus*, *Tripyla*, *Filenchus*, *Pseudacrobeles*, and was positively associated with *Meloidogyne*, *Xenocriconemella*, and *Teratocephalus* during May 2018 (Fig. 4). SIMPER analysis revealed that the average dissimilarity of the nematode communities increased between the control and other treatments with increasing physical disturbance during January and May 2018 (January 2018: control vs. SLR = 43.2%, control vs. R2M = 49.5%, control vs. R2W = 49%; May 2018: control vs. SLR = 40.2%, control vs. R2M = 42.2%, control vs. R2W = 56.3%). According to the SIMPER test, *Filenchus* contributed most to the dissimilarity followed by *Xenocriconemella, Gracilacus, Plectus, Pseudacrobeles, Ditylenchus, Meloidogyne, Prismatolaimus, Helicotylenchus*, and *Acrobeloides* between the control and other treatments during January 2018. Similarly, *Meloidogyne* contributed most to the dissimilarity followed by *Filenchus, Xenocriconemella, Acrobeles, Gracilacus, Helicotylenchus, Alaimus*, Rhabditidae, *Acrobeloides, Plectus, Prismatolaimus*, and *Ceratoplectus* between the control and other treatments during May 2018 (Supplementary Table 5).

## Discussion

### Increasing levels of physical disturbance on nematode abundance and community composition

Nematodes play a key role in maintaining and regulating several biological processes, crucial for soil and plant health (Yeates and Coleman, 1982; Liang *et al*., 2009). Tillage is one of the most intensively used agricultural management strategies, affecting the most important players in soil biological processes such as decomposition, mineralization, and nutrient cycling (Stinner *et al*., 1984; Dick *et al*., 1988; Fraser *et al*., 1994). Many studies have been conducted to evaluate the effect of different types of physical disturbances on nematode communities and other soil organisms in agricultural ecosystems (Lenz and Eisenbeis, 2000; Okada and Harada, 2007; Rahman *et al*., 2007; Dong *et al*., 2013; Forge *et al*., 2015; Sánchez-Moreno *et al*., 2015; Zhang *et al*., 2015, 2019; Zhong *et al*., 2017; Wu *et al*., 2021). However, this report is the first on the effect of tillage in terms of increasing levels of physical disturbance on nematode populations in a previously undisturbed forest ecosystem.

Tillage significantly reduced the overall abundance and overall richness of nematode communities over time in R2M and R2W, which was attributed to the decrease in the abundance of bacterial feeders, fungal feeders, predators, and omnivores and a decrease in the richness of bacterial feeders, predators, and omnivores. Tillage directly affects nematode communities by abrasion and indirectly by changing the food web, temperature, moisture, and aeration of soil in tillage treatments compared to the control, which was undisturbed (Kladivko, 2001; Holland, 2004; Rahman *et al*., 2007; Golabi *et al*., 2014). Our findings are in agreement with the studies conducted by Freckman and Ettema, (1993), Fu *et al*. (2000), Okada and Harada, (2007), Treonis *et al*. (2010), Dong *et al*. (2013), Sánchez-Moreno *et al*. (2015), Zhang *et al*. (2015), Zhong *et al*. (2017), and Pothula *et al*. (2019), who reported that physical disturbances reduced nematode abundance in agricultural ecosystems. However, it should be noted that our tillage regime was far more intense than that of any agricultural system (1 or 2 times/monthly vs. 1 or 2 times yearly). In some studies, the effect of tillage in agricultural ecosystems was noticed immediately after the first tillage (Lenz and Eisenbeis, 2000); however, in our study, the tillage effect was significantly evident after 9 mon. This may be due to the differences in forest and agricultural ecosystems and their response to tillage regimes. Removal of surface litter, SLR, resulted in a significant decrease in the overall richness of nematodes compared to control during January 2018. Our outcomes are in agreement with the study conducted by Wu *et al*. (2021). However, the significance was not evident during the subsequent sampling. On the other hand, the higher overall abundance and overall richness of nematodes in the control treatment could be attributed to the large amount of litter content and the absence of physical disturbances in the control treatment.

Among nematode trophic groups, tillage significantly reduced the abundance and richness of bacterial feeders over time. Many studies conducted in agricultural fields have reported that tillage stimulated the bacterial feeding nematodes due to the probable increase in bacterial biomass with the incorporation of organic matter (Andren and Lagerlof, 1983; Parmelee and Alston, 1986; Ettema and Bongers, 1993; Lenz and Eisenbeis, 2000; Liphadzi *et al*., 2005; Sánchez-Moreno *et al*., 2006). However, the decrease in bacterial feeders in our study was attributed to the decrease in the abundance of *Acrobeles*, *Alaimus, Ceratoplectus*, and *Prismatolaimus*. Moreover, the decrease in bacterial feeders was also attributed to the declining trend of the abundance of *Plectus* and *Pseudacrobeles*, even though the difference was not statistically significant. This may be due to the fact that organic litter was periodically removed from the tillage treatments. Similarly, several studies have reported that *Prismatolaimus* is reduced by cultivation (Fiscus and Neher, 2002; Ferris and Bongers, 2006; Minoshima *et al*., 2007; Sánchez-Moreno *et al*., 2009; Zhao and Neher, 2013; Zhang *et al*., 2019).

Tillage significantly reduced the abundance but not the richness of fungal feeders. There is a discrepancy in reports on the response of fungal feeding nematodes to tillage practices. Some studies have reported that tillage increased the fungal feeding nematode communities (Parmelee and Alston, 1986; Liphadzi *et al*., 2005; Sánchez-Moreno *et al*., 2006; Dong *et al*., 2013). However, Okada and Harada (2007) found that fungal-feeding nematodes increased in a no-till system. This discrepancy may be due to a complex set of factors, including geographic location, type of vegetation, soil type, and ecosystem. Unlike the case in agricultural ecosystems, tillage decreased the abundance of *Filenchus* in our study. Studies conducted in agriculture ecosystems have noted that *Filenchus* has an excellent capacity to tolerate disturbances and occurs in soils with abundant organic matter (Fiscus and Neher, 2002; Okada and Kadota, 2003; Zhang *et al*., 2015, 2019). This disagreement may be due to the condition of the ecosystem that was undisturbed and plausibly due to the consistent removal of surface litter in our study.

Among nematodes belonging to the higher hierarchy of the soil food web, tillage significantly reduced the abundance and richness of predators and omnivores, which are sensitive to disturbances (Bongers 1990; Ferris *et al*., 2001). Similar results were reported by Dong *et al*. (2013), Zhang *et al*. (2015), and Zhang *et al*. (2019). The tillage treatments negatively impacted the abundance of *Aporcelaimellus, Clarkus*, Dorylaimida, and *Tripyla*, resulting in the decline of predators and omnivores. The sensitivity of *Aporcelaimellus* to tillage was previously reported in the agricultural ecosystem by Zhong *et al*. (2016, 2017). Similarly, Dorylaimida was also reported to be lowered by cultivation (Fiscus and Neher, 2002; Rahman *et al*., 2007; Zhang *et al*., 2012). Fiscus and Neher (2002) reported that *Clarkus* is tolerant to direct effects of tillage in agricultural ecosystems; however, in our study, the abundance of *Clarkus* was reduced with increasing levels of physical disturbance. This incongruity probably resulted as our study was carried out in an undisturbed forest ecosystem, where nematodes were not previously exposed to any physical disturbances and therefore may not have been selected for it. Unlike other trophic groups, the abundance and richness of plant-feeding nematodes did not differ significantly in tillage treatments compared to control. The response of plant-feeding nematodes to tillage practices is complicated to interpret as they are more closely associated with plants than with soil (Sánchez-Moreno *et al*., 2006).

The effect of tillage disturbances on nematode communities according to c-p classes was also assessed. The abundance and richness of c-p 2, c-p 4, and c-p 5 class nematodes were significantly decreased by tillage. Nematodes belonging to lower c-p classes are r-strategists, which are characterized by a high fecundity rate, short generation time, and tolerance to disturbances (Bongers, 1990; Ferris *et al*., 2001). Although c-p 2 class nematodes belong to lower c-p classes, the abundance and richness of c-p 2 class nematodes were significantly reduced by tillage. The decrease in c-p 2 class nematodes in tillage treatments was due to the decrease in the abundance of *Acrobeles*, *Boleodorus*, *Ceratoplectus*, and *Filenchus*. The abundance and richness of nematodes of higher c-p classes (c-p 4 and c-p 5) were significantly reduced by tillage disturbances as these nematodes are sensitive to disturbances in the soil ecosystem (Bongers, 1990; Lenz and Eisenbeis, 2000; Ferris *et al*., 2001).

The significant differences between the control and SLR were not reflected in trophic and c-p group analyses. However, individual nematode analyses revealed that removal of surface litter resulted in a significant decrease in the abundance of *Acrobeles*, *Aporcelaimellus*, and *Boleodorus*. These three genera belong to different trophic groups: bacterial feeder, omnivore, and plant feeder, respectively. Further research at the genus level is needed to explain the impact of surface litter on different nematode genera. Overall, these results indicate that the sensitivity of individual nematode genera to increasing levels of physical disturbances is different, and the sensitivity should be considered to foresee the function of soil organisms in the soil.

### Increasing levels of physical disturbance on nematode ecological indices

The nematode ecological indices are often used to assess the condition of the soil food web. l and H´ indicate the diversity of nematode communities. Increasing levels of physical disturbance increased the Simpson index (l) and decreased the Shannon diversity index (H´). This agreement indicates the decrease in diversity of nematode communities with the increase in the level of physical disturbance. The NCR value decreased with increasing levels of physical disturbance, indicating the shift of the decomposition pathway from bacterial to fungal dominated. The higher value of NCR in the control treatment suggested the predominance of the bacterial decomposition pathway. Our results are in agreement with Zhang *et al*., 2015 but not in concurrence with Okada and Harada, 2007 and Treonis *et al*., 2010, who reported a fungal-dominated decomposition channel in no-tillage. Our results indicated that the change in the decomposition channel may be due to the continuous removal of surface litter, which reduced the surface organic matter and shifted the decomposition channel from bacterial to fungal dominated. MI and PPI are used to assess the effect of disturbances on soil food webs. Our results showed that tillage significantly decreased MI values in both R2W and R2M compared to the control and SLR treatments during the last sampling in May 2018. On the other hand, the removal of surface litter did not affect the MI value. This indicates a lower abundance and diversity of higher c-p class nematodes in tillage treatments due to heightened levels of physical disturbances (Djigal *et al*., 2012; Grabau and Chen, 2016; Zhong *et al*., 2016; Zhong *et al*., 2017). However, tillage did not significantly affect PPI values as the abundance of plant feeders was not affected much in the tillage treatments. Our results suggested that the EI value significantly increased with the increasing levels of physical disturbance. Although the abundance of bacterial feeders belonging to the c-p 2 class declined drastically, the abundance of enrichment opportunists (c-p 1 nematodes) slightly decreased with the increase in levels of physical disturbance, which resulted in higher EI values in tillage treatments. In contrast, SI significantly decreased with the increasing levels of physical disturbance. The lower SI values in tillage treatments indicate that the ecosystem was disturbed with fewer predators and omnivores, which are sensitive to disturbances (Korthals *et al*., 1996; Ferris *et al*., 2001). Similar results were reported by Sánchez-Moreno *et al*. (2009) and Zhang *et al*. (2015).

### Increasing levels of physical disturbance on nematode FMF

Nematode FMF were calculated to indicate the structure and function of soil food webs with different levels of physical disturbance. The nematode trophic footprints suggested the changes in the metabolic activity and flow of C into the soil food web through their respective trophic channels (Ferris, 2010). The value of efoot is considered as an indicator of the flow of C and energy through r-strategists, which are lower c-p values (1–2) (Ferris *et al*., 2012). The value of sfoot indicates the flow of C and energy through higher c-p values (3–5), which may regulate the function of soil food webs (Neher *et al*., 2004; Ferris *et al*., 2012). The higher value of efoot in the control treatment indicated higher productivity and turnover rates of the r-strategists and adequate resources (Vonk *et al*., 2013; Ito *et al*., 2015; Zhang *et al*., 2015; Zhong *et al*., 2017). Similarly, tillage drastically reduced the values of sfoot, which indicates the decrease in metabolic activity of both predators and omnivores in R2M and R2W treatments (Zhong *et al*., 2016).

The FMF area of nematode communities decreased with increasing levels of physical disturbance. FMF with a larger area in the control treatment during May 2018 indicated a higher metabolic activity and inflow of C, which was used for nematode production (Ferris, 2010). The high availability of organic matter in the control treatment increased the abundance of predators and omnivores and activated a stronger pathway through the predator channel, which may promote the stronger metabolic process and stability of the soil food web (Ferris, 2010; Thakur and Geisen, 2019; Kou *et al*., 2020). On the other hand, the FMF area was smaller in tillage treatments, indicating that smaller quantities of C were used for nematode production and lower metabolic activity due to the low availability of resources and lower predator-omnivore numbers (Ferris, 2010). All the treatments were clustered together in the same quadrat until November 2017 and started spreading out in January 2018. In May 2018, control and SLR treatments were located in quadrat B, which indicated a maturing ecosystem with enriched soil nutrients and a well-structured soil food web, while R2M and R2W were located in quadrat A, which indicated a disturbed and poorly structured soil food web (Ferris, 2010).

### The relationship between nematode abundance and increasing levels of physical disturbances

Soil nematodes have been used as bioindicators to assess the effect of physical disturbances (Yeates, 2003). Our study revealed that different nematode genera had varying sensitivities to the physical disturbances. NMDS analysis of nematode communities revealed that soil nematode genera were clearly separated by increasing levels of physical disturbance during January and May 2018. The dissimilarity between the treatments indicates the progressive decrease in the abundance of nematode communities at R2M and R2W treatments. These declining trends generated significantly different nematode assemblages at all treatments during January and May 2018, as emphasized by PERMANOVA. All the nematodes belonging to higher c-p classes (c-p 4 and 5) and bacterial feeders of the c-p 2 class were negatively affected by the R2M and R2W treatments, while nematodes of lower c-p classes including bacterial feeders except c-p 2 class, fungal feeders, and plant feeders were not impacted by the tillage treatments. Nematodes of higher c-p classes such as Dorylaimida, *Aporcelaimellus*, *Alaimus*, *Clarkus*, and *Tripyla* were sensitive to physical disturbances (Bongers, 1990; Lenz and Eisenbeis, 2000; Ferris *et al*., 2001). Although bacterial feeders of the c-p 2 class belong to lower c-p classes, the abundance of c-p 2 class nematodes such as *Tylocephalus*, *Acrobeles*, *Ceratoplectus, Plectus, and Pseudacrobeles* was significantly reduced by tillage. The decrease in c-p 2 class nematodes in tillage treatments may be due to the continuous removal of organic matter (Ferris and Bongers, 2006). Tillage negatively impacted only a few plant feeders such as *Helicotylenchus*, *Gracilacus*, and *Boleodorus*. This declining trend of plant-feeding nematodes was in agreement with Lenz and Eisenbeis (2000) and Rahman *et al*. (2007). However, *Meloidogyne*, *Xenocriconemella*, and *Ecphyadophora* are positively associated with tillage. The response of plant-feeding nematodes to tillage practices is complicated to interpret as they are more closely associated with plants than with soil (Sanchez-Moreno *et al*., 2006). Furthermore, SIMPER analysis also revealed that the above-mentioned genera lead to significant dissimilarity among treatments.

Overall, this study gives an insight into the effect of increasing levels of physical disturbance on nematode communities in an undisturbed forest ecosystem, indicating that tillage reduced the abundance and richness of nematode communities, which was consistent with previous studies in the literature that were conducted in agricultural ecosystems. However, in this study, bacterial feeding nematodes belonging to the c-p 2 class responded differently compared to those of agricultural ecosystems. Tillage significantly reduced the abundance and richness of bacterial feeding nematodes of the c-p 2 class along with predators and omnivores, which belong to higher c-p classes. Moreover, tillage significantly reduced the FMF of nematodes, which indicates decreased metabolic activity, reduced C inflow, and poorly structured soil food webs. Unlike tillage, minimal disturbance such as removal of surface litter resulted in a significant reduction of very few nematode genera. Previous studies conducted in agricultural ecosystems determined that *Clarkus*, *Filenchus*, and *Plectus* were tolerant to tillage; however, they were found sensitive to tillage in our study. To understand this incongruity, further studies are needed to investigate whether these species are adapted to the physical disturbances in agricultural ecosystems. Overall, our study suggests that increasing levels of physical disturbance are detrimental to nematode community abundance and diversity that could affect ecosystem stability and sustainability. Also, our results affirmed that soil nematodes are highly sensitive to physical disturbances and therefore could be used as indicators of stability and functioning of the soil ecosystem.

## Figures and Tables

**Table S1:** Abundance (individuals per 100 cm^3^ of soil) of nematode taxon in different treatments: control, SLR, R2M, and R2W at all sampling times. (mean ± pooled SE, *n* = 5).

Taxon	Abbreviation	Trophic groups	Time	Control	SLR	R2M	R2W
Rhabditidae	Rhab	BF	Apr-17	115.67 ± 34.19	96 ± 34.19	177.67 ± 34.19	96.67 ± 34.19
			Jun-17	147.67 ± 34.19	57 ± 34.19	56.33 ± 34.19	61.67 ± 34.19
			Sep-17	134.67 ± 34.19	120 ± 34.19	93.67 ± 34.19	30 ± 34.19
			Nov-17	30.67 ± 34.19	46.67 ± 34.19	60.67 ± 34.19	20.67 ± 34.19
			Jan-18	22 ± 34.19	27.33 ± 34.19	15.33 ± 34.19	23.33 ± 34.19
			May-18	46 ± 34.19	34 ± 34.19	33.33 ± 34.19	21 ± 34.19
*Plectus*	Plec	BF	Apr-17	55.67 ± 11.23	26.33 ± 11.23	29.33 ± 11.23	30.33 ± 11.23
			Jun-17	72.33 ± 11.23	40 ± 11.23	16.33 ± 11.23	20.33 ± 11.23
			Sep-17	50 ± 11.23	63 ± 11.23	10.67 ± 11.23	17.67 ± 11.23
			Nov-17	29.67 ± 11.23	22 ± 11.23	11.67 ± 11.23	12.67 ± 11.23
			Jan-18	40.67 ± 11.23	21 ± 11.23	10.33 ± 11.23	5.67 ± 11.23
			May-18	45.67 ± 11.23	24.67 ± 11.23	28 ± 11.23	6.67 ± 11.23
*Acrobeloides*	Acrd	BF	Apr-17	46.33 ± 12.83	53.67 ± 12.83	24 ± 12.83	30 ± 12.83
			Jun-17	18.67 ± 12.83	27 ± 12.83	28.33 ± 12.83	19 ± 12.83
			Sep-17	11.67 ± 12.83	31.33 ± 12.83	36.33 ± 12.83	37.67 ± 12.83
			Nov-17	14.67 ± 12.83	11.67 ± 12.83	15 ± 12.83	18 ± 12.83
			Jan-18	23.33 ± 12.83	22 ± 12.83	18.67 ± 12.83	18.33 ± 12.83
			May-18	22.33 ± 12.83	18 ± 12.83	32.67 ± 12.83	14 ± 12.83
*Prismatolaimus*	Pris	BF	Apr-17	7 ± 10.29	2.67 ± 10.29	2.33 ± 10.29	2.67 ± 10.29
			Jun-17	19 ± 10.29	28 ± 10.29	6 ± 10.29	12.33 ± 10.29
			Sep-17	28.33 ± 10.29	37.67 ± 10.29	20.67 ± 10.29	41.67 ± 10.29
			Nov-17	43.67 ± 10.29	37.33 ± 10.29	24.67 ± 10.29	13.67 ± 10.29
			Jan-18	24.67 ± 10.29^a^	19.33 ± 10.29^a,b^	6 ± 10.29^a,b^	1 ± 10.29^b^
			May-18	21.67 ± 10.29^a^	30.67 ± 10.29^a,b^	10 ± 10.29^a,b^	1.67 ± 10.29^b^
*Pseudacrobeles*	Pseu	BF	Apr-17	20 ± 5.28	22 ± 5.28	11.67 ± 5.28	11.33 ± 5.28
			Jun-17	18.67 ± 5.28	5.67 ± 5.28	6 ± 5.28	6.67 ± 5.28
			Sep-17	19.33 ± 5.28	8 ± 5.28	8.67 ± 5.28	1 ± 5.28
			Nov-17	15.67 ± 5.28	4.33 ± 5.28	10.33 ± 5.28	9.33 ± 5.28
			Jan-18	37.33 ± 5.28	2 ± 5.28	8.33 ± 5.28	12.33 ± 5.28
			May-18	14.67 ± 5.28	4 ± 5.28	2.67 ± 5.28	3 ± 5.28
*Alaimus*	Alai	BF	Apr-17	0 ± 8.85	0 ± 8.85	0 ± 8.85	0 ± 8.85
			Jun-17	10.33 ± 8.85	5.33 ± 8.85	6.33 ± 8.85	0 ± 8.85
			Sep-17	33.67 ± 8.85	13 ± 8.85	20 ± 8.85	16 ± 8.85
			Nov-17	31 ± 8.85	76.33 ± 8.85	44.33 ± 8.85	15.33 ± 8.85
			Jan-18	10 ± 8.85	8 ± 8.85	0.67 ± 8.85	3 ± 8.85
			May-18	24.33 ± 8.85^a^	38 ± 8.85^a^	6.67 ± 8.85^b^	0 ± 8.85^b^
*Acrobeles*	Acrb	BF	Apr-17	6.67 ± 7.02	2.67 ± 7.02	1.67 ± 7.02	1.33 ± 7.02
			Jun-17	1.67 ± 7.02	6.33 ± 7.02	5.67 ± 7.02	7.67 ± 7.02
			Sep-17	7.67 ± 7.02	12.33 ± 7.02	12.33 ± 7.02	2 ± 7.02
			Nov-17	21 ± 7.02	6 ± 7.02	12 ± 7.02	1 ± 7.02
			Jan-18	2.67 ± 7.02	16.67 ± 7.02	5.67 ± 7.02	0 ± 7.02
			May-18	32.67 ± 7.02^a^	9.67 ± 7.02^b^	5.67 ± 7.02^b^	0 ± 7.02^b^
*Tylocephalus*	Tylo	BF	Apr-17	16.67 ± 2.7	4.67 ± 2.7	9.67 ± 2.7	5 ± 2.7
			Jun-17	9.67 ± 2.7	7.67 ± 2.7	0.67 ± 2.7	6.33 ± 2.7
			Sep-17	5.67 ± 2.7	3.33 ± 2.7	1.33 ± 2.7	1 ± 2.7
			Nov-17	2.33 ± 2.7	1.33 ± 2.7	0 ± 2.7	3 ± 2.7
			Jan-18	9 ± 2.7	2.33 ± 2.7	0 ± 2.7	0.67 ± 2.7
			May-18	3.33 ± 2.7	0.67 ± 2.7	1 ± 2.7	0 ± 2.7
*Ceratoplectus*	Cera	BF	Apr-17	3 ± 2.79	2.67 ± 2.79	2.33 ± 2.79	1 ± 2.79
			Jun-17	5 ± 2.79	12.33 ± 2.79	7.33 ± 2.79	5.33 ± 2.79
			Sep-17	9.33 ± 2.79	5.33 ± 2.79	5 ± 2.79	1 ± 2.79
			Nov-17	8.33 ± 2.79	8.33 ± 2.79	1 ± 2.79	0 ± 2.79
			Jan-18	7 ± 2.79^a^	1.33 ± 2.79^a,b^	0 ± 2.79^b^	0 ± 2.79^b^
			May-18	15 ± 2.79^a^	3.33 ± 2.79^a,b^	1 ± 2.79^b^	0 ± 2.79^b^
*Teratocephalus*	Tera	BF	Apr-17	3 ± 2.62	1.67 ± 2.62	2.33 ± 2.62	0 ± 2.62
			Jun-17	0 ± 2.62	1.67 ± 2.62	0.67 ± 2.62	0 ± 2.62
			Sep-17	12.33 ± 2.62	7 ± 2.62	2.33 ± 2.62	2 ± 2.62
			Nov-17	3.33 ± 2.62	2.67 ± 2.62	0 ± 2.62	0 ± 2.62
			Jan-18	5.33 ± 2.62	2 ± 2.62	0.67 ± 2.62	0.67 ± 2.62
			May-18	1.33 ± 2.62	0 ± 2.62	0.67 ± 2.62	2.67 ± 2.62
*Cervidellus*	Cerv	BF	Apr-17	1 ± 1.61	0 ± 1.61	1 ± 1.61	1 ± 1.61
			Jun-17	0 ± 1.61	0 ± 1.61	0.67 ± 1.61	0.67 ± 1.61
			Sep-17	2.33 ± 1.61	3.33 ± 1.61	1 ± 1.61	3 ± 1.61
			Nov-17	1.67 ± 1.61	1 ± 1.61	1 ± 1.61	2.33 ± 1.61
			Jan-18	5.67 ± 1.61	1.33 ± 1.61	2 ± 1.61	1 ± 1.61
			May-18	5 ± 1.61	0.67 ± 1.61	3.33 ± 1.61	0 ± 1.61
*Diplogasteridae*	Dipl	BF	Apr-17	0 ± 1.21	0 ± 1.21	1.67 ± 1.21	0 ± 1.21
			Jun-17	1.67 ± 1.21	0 ± 1.21	0.67 ± 1.21	2.67 ± 1.21
			Sep-17	2.67 ± 1.21	3.67 ± 1.21	0 ± 1.21	0 ± 1.21
			Nov-17	1 ± 1.21	0 ± 1.21	1.67 ± 1.21	0 ± 1.21
			Jan-18	2.33 ± 1.21	0 ± 1.21	0.67 ± 1.21	0 ± 1.21
			May-18	2.33 ± 1.21	0 ± 1.21	0 ± 1.21	1 ± 1.21
*Filenchus*	File	FF	Apr-17	55.33 ± 26.02	52.33 ± 26.02	65.67 ± 26.02	113.67 ± 26.02
			Jun-17	158.67 ± 26.02	111.67 ± 26.02	75.67 ± 26.02	94.67 ± 26.02
			Sep-17	116.33 ± 26.02	196.33 ± 26.02	133 ± 26.02	123.33 ± 26.02
			Nov-17	106 ± 26.02	130.33 ± 26.02	142.67 ± 26.02	63.67 ± 26.02
			Jan-18	191.33 ± 26.02^a^	156.67 ± 26.02^a^,b	93.33 ± 26.02^b^	88.33 ± 26.02^b^
			May-18	130.67 ± 26.02^a^	76.67 ± 26.02^a^,b	49.33 ± 26.02^b^	25 ± 26.02^b^
*Aphelenchoides*	Aphe	FF	Apr-17	33.33 ± 9.81	26.67 ± 9.81	48.33 ± 9.81	27.67 ± 9.81
			Jun-17	29.33 ± 9.81	20.33 ± 9.81	12 ± 9.81	11.33 ± 9.81
			Sep-17	36 ± 9.81	48 ± 9.81	18 ± 9.81	16.67 ± 9.81
			Nov-17	17 ± 9.81	27 ± 9.81	16.33 ± 9.81	21.33 ± 9.81
			Jan-18	25.33 ± 9.81	9.33 ± 9.81	9.67 ± 9.81	16.33 ± 9.81
			May-18	15 ± 9.81	5.67 ± 9.81	23.33 ± 9.81	16.67 ± 9.81
*Ditylenchus*	Dity	FF	Apr-17	0 ± 5.13	0 ± 5.13	0 ± 5.13	0 ± 5.13
			Jun-17	16.67 ± 5.13	2.33 ± 5.13	5 ± 5.13	5 ± 5.13
			Sep-17	24.67 ± 5.13	4.33 ± 5.13	15.33 ± 5.13	13.33 ± 5.13
			Nov-17	21.33 ± 5.13	4.33 ± 5.13	10.67 ± 5.13	26.33 ± 5.13
			Jan-18	28 ± 5.13	9 ± 5.13	19.67 ± 5.13	13.67 ± 5.13
			May-18	24.33 ± 5.13	4.33 ± 5.13	9.67 ± 5.13	8.67 ± 5.13
*Diphtherophora*	Diph	FF	Apr-17	0 ± 3.54	0 ± 3.54	0 ± 3.54	0 ± 3.54
			Jun-17	13.33 ± 3.54	9 ± 3.54	0 ± 3.54	5 ± 3.54
			Sep-17	1.67 ± 3.54	8 ± 3.54	1.33 ± 3.54	3.67 ± 3.54
			Nov-17	6.67 ± 3.54	5 ± 3.54	4 ± 3.54	2 ± 3.54
			Jan-18	2.33 ± 3.54	1 ± 3.54	0 ± 3.54	0.67 ± 3.54
			May-18	2.67 ± 3.54	4.33 ± 3.54	0.67 ± 3.54	2.33 ± 3.54
*Meloidogyne*	Melo	PF	Apr-17	126.33 ± 31.95	191.33 ± 31.95	135 ± 31.95	104.33 ± 31.95
			Jun-17	165.33 ± 31.95	104.33 ± 31.95	46.33 ± 31.95	40.33 ± 31.95
			Sep-17	66.67 ± 31.95	97 ± 31.95	76 ± 31.95	33.67 ± 31.95
			Nov-17	47.33 ± 31.95	58.67 ± 31.95	51.67 ± 31.95	55 ± 31.95
			Jan-18	29.67 ± 31.95	42 ± 31.95	10.33 ± 31.95	16.33 ± 31.95
			May-18	135.33 ± 31.95	140.67 ± 31.95	190.67 ± 31.95	114.33 ± 31.95
*Xenocriconemella*	Xeno	PF	Apr-17	16 ± 26.89	8 ± 26.89	6.33 ± 26.89	29.33 ± 26.89
			Jun-17	29 ± 26.89	30.67 ± 26.89	9 ± 26.89	40.67 ± 26.89
			Sep-17	63.33 ± 26.89	41.67 ± 26.89	16 ± 26.89	92 ± 26.89
			Nov-17	95 ± 26.89	112 ± 26.89	38 ± 26.89	80.67 ± 26.89
			Jan-18	57.67 ± 26.89	108.67 ± 26.89	16.33 ± 26.89	93.67 ± 26.89
			May-18	18 ± 26.89	46.67 ± 26.89	10 ± 26.89	47.33 ± 26.89
*Gracilacus*	Grac	PF	Apr-17	11 ± 22.18	14 ± 22.18	8.67 ± 22.18	12.67 ± 22.18
			Jun-17	19.67 ± 22.18	13.67 ± 22.18	2 ± 22.18	12.67 ± 22.18
			Sep-17	24 ± 22.18	77 ± 22.18	17.33 ± 22.18	28.33 ± 22.18
			Nov-17	58.67 ± 22.18	64 ± 22.18	21.67 ± 22.18	72 ± 22.18
			Jan-18	49 ± 22.18	42.33 ± 22.18	11 ± 22.18	14.67 ± 22.18
			May-18	28.33 ± 22.18	4.33 ± 22.18	4.67 ± 22.18	3.67 ± 22.18
*Helicotylenchus*	Heli	PF	Apr-17	6.67 ± 18.12	7.67 ± 18.12	2.67 ± 18.12	1.33 ± 18.12
			Jun-17	20.67 ± 18.12	8.67 ± 18.12	2.33 ± 18.12	7.33 ± 18.12
			Sep-17	6.67 ± 18.12	74.67 ± 18.12	1 ± 18.12	2 ± 18.12
			Nov-17	56.67 ± 18.12	6 ± 18.12	1 ± 18.12	3 ± 18.12
			Jan-18	23.33 ± 18.12	2.33 ± 18.12	0.67 ± 18.12	1 ± 18.12
			May-18	22.33 ± 18.12	3.67 ± 18.12	0 ± 18.12	3.67 ± 18.12
*Boleodorus*	Bole	PF	Apr-17	0 ± 3.85	0 ± 3.85	0 ± 3.85	0 ± 3.85
			Jun-17	10 ± 3.85	10.67 ± 3.85	7.33 ± 3.85	6.67 ± 3.85
			Sep-17	13.33 ± 3.85	1 ± 3.85	10.33 ± 3.85	6 ± 3.85
			Nov-17	4 ± 3.85	14 ± 3.85	11.33 ± 3.85	1 ± 3.85
			Jan-18	6.33 ± 3.85	0 ± 3.85	0 ± 3.85	0 ± 3.85
			May-18	12.67 ± 3.85^a^	3 ± 3.85^b^	0 ± 3.85^b^	0 ± 3.85^b^
*Ecphyadophora*	Ecph	PF	Apr-17	1 ± 1.23	0 ± 1.23	0 ± 1.23	0 ± 1.23
			Jun-17	0 ± 1.23	3.33 ± 1.23	1.33 ± 1.23	0.67 ± 1.23
			Sep-17	5.33 ± 1.23	0 ± 1.23	0 ± 1.23	0 ± 1.23
			Nov-17	2.33 ± 1.23	1 ± 1.23	1 ± 1.23	1.67 ± 1.23
			Jan-18	6.67 ± 1.23	0 ± 1.23	3.33 ± 1.23	0 ± 1.23
			May-18	1.33 ± 1.23	0 ± 1.23	1.67 ± 1.23	1 ± 1.23
*Clarkus*	Clak	PR	Apr-17	0 ± 2.13	0.67 ± 2.13	3.67 ± 2.13	0 ± 2.13
			Jun-17	3 ± 2.13	2.33 ± 2.13	0.67 ± 2.13	0 ± 2.13
			Sep-17	2.67 ± 2.13	1.67 ± 2.13	4 ± 2.13	0 ± 2.13
			Nov-17	3.33 ± 2.13	6.67 ± 2.13	7.67 ± 2.13	3.67 ± 2.13
			Jan-18	3.33 ± 2.13	0 ± 2.13	1.33 ± 2.13	0 ± 2.13
			May-18	5.67 ± 2.13^a^	1.67 ± 2.13^a,b^	0 ± 2.13^b^	0 ± 2.13^b^
*Tripyla*	Trip	PR	Apr-17	0 ± 1.93	0 ± 1.93	0 ± 1.93	0 ± 1.93
			Jun-17	1.67 ± 1.93	1.33 ± 1.93	0.67 ± 1.93	0.67 ± 1.93
			Sep-17	3.67 ± 1.93	2.33 ± 1.93	0 ± 1.93	0 ± 1.93
			Nov-17	5 ± 1.93	5.33 ± 1.93	2 ± 1.93	0 ± 1.93
			Jan-18	0 ± 1.93	0 ± 1.93	0 ± 1.93	0 ± 1.93
			May-18	5 ± 1.93^a^	2.33 ± 1.93^a,b^	0 ± 1.93^b^	0 ± 1.93^b^
Dorylaimida	Dory	OM	Apr-17	3.67 ± 3.33	7.67 ± 3.33	0 ± 3.33	14.33 ± 3.33
			Jun-17	0 ± 3.33	0 ± 3.33	0.67 ± 3.33	0.67 ± 3.33
			Sep-17	3.33 ± 3.33	5.33 ± 3.33	4.33 ± 3.33	10.67 ± 3.33
			Nov-17	11.67 ± 3.33	13.67 ± 3.33	2.67 ± 3.33	5.33 ± 3.33
			Jan-18	2.33 ± 3.33	0 ± 3.33	0 ± 3.33	0 ± 3.33
			May-18	8.67 ± 3.33^a^	7.33 ± 3.33^a,b^	0 ± 3.33^b^	0 ± 3.33^b^
*Aporcelaimellus*	Apor	OM	Apr-17	0 ± 2	0.67 ± 2	0 ± 2	0 ± 2
			Jun-17	4.67 ± 2	2.33 ± 2	1.33 ± 2	1.33 ± 2
			Sep-17	3 ± 2	2.33 ± 2	3.67 ± 2	2 ± 2
			Nov-17	4 ± 2	7.33 ± 2	5.67 ± 2	3.67 ± 2
			Jan-18	4 ± 2	2.33 ± 2	2 ± 2	0 ± 2
			May-18	6 ± 2^a^	1.67 ± 2^b^	0 ± 2^b^	0 ± 2^b^

Letters indicate significant differences among treatments at each sampling time at *P <* 0.05 (Tukey-LSD test). OM, omnivores; PF, plant feeders; PR, predators; R2M, rototill for every 2 mon; R2W, rototill for every 2 wk; SLR, surface litter removed.

**Table S2:** Abundance and richness of nematode trophic groups per 100 cm^3^ of soil in different treatments: control, SLR, R2M, and R2W at all sampling times. (mean ± pooled SE, *n* = 5).

Trophic group	Time	Control	SLR	R2M	R2W
**Abundance**					
BF	Apr-17	274.33 ± 54.05	212.67 ± 54.05	264.67 ± 54.05	180 ± 54.05
	Jun-17	305.33 ± 54.05	191.33 ± 54.05	135 ± 54.05	145 ± 54.05
	Sep-17	318.67 ± 54.05	311.33 ± 54.05	212.67 ± 54.05	154 ± 54.05
	Nov-17	206 ± 54.05	220.67 ± 54.05	183 ± 54.05	97 ± 54.05
	Jan-18	195.67 ± 54.05	130 ± 54.05	73.67 ± 54.05	66.33 ± 54.05
	May-18	257.67 ± 54.05^a^	174 ± 54.05^a,b^	125.67 ± 54.05^a,b^	50 ± 54.05^b^
FF	Apr-17	88.33 ± 32.47	79.67 ± 32.47	114 ± 32.47	141.33 ± 32.47
	Jun-17	218.33 ± 32.47	143.67 ± 32.47	92.67 ± 32.47	116.33 ± 32.47
	Sep-17	181 ± 32.47	264 ± 32.47	167 ± 32.47	157.33 ± 32.47
	Nov-17	158.67 ± 32.47	169.67 ± 32.47	173.67 ± 32.47	114.67 ± 32.47
	Jan-18	249 ± 32.47^a^	175.33 ± 32.47^a,b^	122.67 ± 32.47^b^	119.33 ± 32.47^b^
	May-18	189.67 ± 32.47^a^	100 ± 32.47^a,b^	84.33 ± 32.47^a,b^	53.33 ± 32.47^b^
PP	Apr-17	170 ± 51.02	225.33 ± 51.02	153 ± 51.02	148.67 ± 51.02
	Jun-17	244.33 ± 51.02	171.67 ± 51.02	68.67 ± 51.02	109.33 ± 51.02
	Sep-17	186.33 ± 51.02	294 ± 51.02	122.67 ± 51.02	163.67 ± 51.02
	Nov-17	271 ± 51.02	259.67 ± 51.02	134 ± 51.02	216.67 ± 51.02
	Jan-18	175 ± 51.02	196 ± 51.02	44.67 ± 51.02	126.67 ± 51.02
	May-18	238.33 ± 51.02	207.67 ± 51.02	208 ± 51.02	169.67 ± 51.02
PR	Apr-17	0 ± 3.75	0.67 ± 3.75	3.67 ± 3.75	4.33 ± 3.75
	Jun-17	4.67 ± 3.75	5 ± 3.75	3.67 ± 3.75	0.67 ± 3.75
	Sep-17	7.67 ± 3.75	4 ± 3.75	5 ± 3.75	2.33 ± 3.75
	Nov-17	11 ± 3.75	14 ± 3.75	17 ± 3.75	4.67 ± 3.75
	Jan-18	3.33 ± 3.75	0 ± 3.75	2 ± 3.75	0 ± 3.75
	May-18	13.33 ± 3.75^a^	5.33 ± 3.75^a,b^	0 ± 3.75^b^	0 ± 3.75^b^
OM	Apr-17	3.67 ± 7.66	8.67 ± 7.66	0 ± 7.66	14.33 ± 7.66
	Jun-17	6.33 ± 7.66	2.33 ± 7.66	2 ± 7.66	5.33 ± 7.66
	Sep-17	7.33 ± 7.66	7.67 ± 7.66	9.33 ± 7.66	12.67 ± 7.66
	Nov-17	18.33 ± 7.66	23.67 ± 7.66	9.67 ± 7.66	13 ± 7.66
	Jan-18	8.33 ± 7.66	2.33 ± 7.66	2.67 ± 7.66	0 ± 7.66
	May-18	48.67 ± 7.66^a^	20.33 ± 7.66^a,b^	0.67 ± 7.66^b^	0 ± 7.66^b^
**Richness**					
BF	Apr-17	7 ± 0.96	6.67 ± 0.96	7.33 ± 0.96	6 ± 0.96
	Jun-17	7.67 ± 0.96	8 ± 0.96	8 ± 0.96	9 ± 0.96
	Sep-17	10.33 ± 0.96	9.67 ± 0.96	9.33 ± 0.96	8.33 ± 0.96
	Nov-17	11 ± 0.96	9 ± 0.96	7 ± 0.96	7.67 ± 0.96
	Jan-18	10.67 ± 0.96^a^	9 ± 0.96^a,b^	8 ± 0.96^a,b^	6.33 ± 0.96^b^
	May-18	10.33 ± 0.96^a^	8.67 ± 0.96^a^	7 ± 0.96^a,b^	4.67 ± 0.96^b^
FF	Apr-17	2 ± 0.41	2 ± 0.41	2 ± 0.41	1.67 ± 0.41
	Jun-17	3 ± 0.41	3 ± 0.41	2.67 ± 0.41	4 ± 0.41
	Sep-17	4 ± 0.41	4 ± 0.41	3.33 ± 0.41	3.67 ± 0.41
	Nov-17	5 ± 0.41	4 ± 0.41	3.67 ± 0.41	3.67 ± 0.41
	Jan-18	4 ± 0.41	3.33 ± 0.41	3 ± 0.41	3.33 ± 0.41
	May-18	4 ± 0.41	4 ± 0.41	4 ± 0.41	3.67 ± 0.41
PP	Apr-17	4.33 ± 0.8	3 ± 0.8	3.33 ± 0.8	3.33 ± 0.8
	Jun-17	4.33 ± 0.8	5 ± 0.8	4.33 ± 0.8	4 ± 0.8
	Sep-17	5.33 ± 0.8	4.67 ± 0.8	4.33 ± 0.8	4.33 ± 0.8
	Nov-17	6.33 ± 0.8	5.33 ± 0.8	5.33 ± 0.8	5.67 ± 0.8
	Jan-18	6 ± 0.8	3.67 ± 0.8	4.33 ± 0.8	3.33 ± 0.8
	May-18	5.67 ± 0.8	4.33 ± 0.8	3 ± 0.8	3.33 ± 0.8
PR	Apr-17	0 ± 0.45	0.33 ± 0.45	0.33 ± 0.45	0.33 ± 0.45
	Jun-17	1 ± 0.45	1 ± 0.45	1.33 ± 0.45	0.33 ± 0.45
	Sep-17	1.67 ± 0.45	0.67 ± 0.45	1 ± 0.45	0.33 ± 0.45
	Nov-17	2 ± 0.45	2 ± 0.45	2.67 ± 0.45	1 ± 0.45
	Jan-18	0.33 ± 0.45	0 ± 0.45	1 ± 0.45	0 ± 0.45
	May-18	1.33 ± 0.45^a,b^	1.67 ± 0.45^a^	0 ± 0.45^b^	0 ± 0.45^b^
OM	Apr-17	0.67 ± 0.42	1 ± 0.42	0 ± 0.42	0.67 ± 0.42
	Jun-17	1 ± 0.42	0.33 ± 0.42	0.67 ± 0.42	2 ± 0.42
	Sep-17	2 ± 0.42	0.67 ± 0.42	1 ± 0.42	1.33 ± 0.42
	Nov-17	2.67 ± 0.42	3.33 ± 0.42	2 ± 0.42	3 ± 0.42
	Jan-18	1.67 ± 0.42	0.33 ± 0.42	1 ± 0.42	0 ± 0.42
	May-18	3.33 ± 0.42^a^	3 ± 0.42^a^	0.33 ± 0.42^b^	0 ± 0.42^b^

Letters indicate significant differences among treatments at each sampling time at *P* < 0.05 (Tukey–LSD test). BF, bacterial feeders; FF, fungal feeders; PP, plant feeders; PR, predators; OM, omnivores; R2M, rototill for every 2 mon; R2W, rototill for every 2 wk; SLR, surface litter removed.

**Table S3:** Abundance and richness of nematode c-p classes per 100 cm^3^ of soil in different treatments: control, SLR, R2M, and R2W at all sampling times. (mean ± SE, *n* = 5).

c-p classes	Time	Control	SLR	R2M	R2W
**Abundance**					
c-p 1	Apr-17	115.67 ± 34.6	97 ± 34.6	179.33 ± 34.6	96.67 ± 34.6
c-p 1	Jun-17	149.33 ± 34.6	57 ± 34.6	57 ± 34.6	65 ± 34.6
c-p 1	Sep-17	137 ± 34.6	123.67 ± 34.6	94.33 ± 34.6	31 ± 34.6
c-p 1	Nov-17	32.33 ± 34.6	46.67 ± 34.6	62.33 ± 34.6	20.67 ± 34.6
c-p 1	Jan-18	25.33 ± 34.6	32.67 ± 34.6	17.67 ± 34.6	23.33 ± 34.6
c-p 1	May-18	51 ± 34.6	35 ± 34.6	33.33 ± 34.6	22 ± 34.6
c-p 2	Apr-17	258.33 ± 51.94	210 ± 51.94	202.33 ± 51.94	235.67 ± 51.94
c-p 2	Jun-17	361 ± 51.94	262.33 ± 51.94	168.67 ± 51.94	197.67 ± 51.94
c-p 2	Sep-17	332.67 ± 51.94	461.33 ± 51.94	268.67 ± 51.94	251.33 ± 51.94
c-p 2	Nov-17	304.67 ± 51.94	295.33 ± 51.94	263 ± 51.94	233.33 ± 51.94
c-p 2	Jan-18	432.33 ± 51.94^a^	283.67 ± 51.94^a,b^	187.67 ± 51.94^b^	172 ± 51.94^b^
c-p 2	May-18	359.67 ± 51.94^a^	158.33 ± 51.94^a,b^	165 ± 51.94^b^	79 ± 51.94^b^
c-p 3	Apr-17	158.67 ± 53.65	211 ± 53.65	150 ± 53.65	137.33 ± 53.65
c-p 3	Jun-17	249.33 ± 53.65	183 ± 53.65	65 ± 53.65	107.33 ± 53.65
c-p 3	Sep-17	184 ± 53.65	272.67 ± 53.65	119 ± 53.65	176 ± 53.65
c-p 3	Nov-17	260.33 ± 53.65	228.67 ± 53.65	121.33 ± 53.65	155 ± 53.65
c-p 3	Jan-18	148 ± 53.65	176.33 ± 53.65	34.67 ± 53.65	114 ± 53.65
c-p 3	May-18	208 ± 53.65	229.67 ± 53.65	212 ± 53.65	171.67 ± 53.65
c-p 4	Apr-17	3.67 ± 11.23	8.67 ± 11.23	3.67 ± 11.23	18.67 ± 11.23
c-p 4	Jun-17	15 ± 11.23	9 ± 11.23	10 ± 11.23	4.67 ± 11.23
c-p 4	Sep-17	44 ± 11.23	21.67 ± 11.23	30.33 ± 11.23	29.33 ± 11.23
c-p 4	Nov-17	56.33 ± 11.23	105.33 ± 11.23	61 ± 11.23	30.67 ± 11.23
c-p 4	Jan-18	19.67 ± 11.23	8 ± 11.23	3.33 ± 11.23	3 ± 11.23
c-p 4	May-18	49 ± 11.23^a^	54 ± 11.23^a^	7.67 ± 11.23^b^	0 ± 11.23^b^
c-p 5	Apr-17	0 ± 6.1	0.67 ± 6.1	0 ± 6.1	0 ± 6.1
c-p 5	Jun-17	4.67 ± 6.1	2.33 ± 6.1	1.33 ± 6.1	1.33 ± 6.1
c-p 5	Sep-17	3 ± 6.1	2.33 ± 6.1	3.67 ± 6.1	2 ± 6.1
c-p 5	Nov-17	11.67 ± 6.1	11.67 ± 6.1	9 ± 6.1	5.33 ± 6.1
c-p 5	Jan-18	6.33 ± 6.1	3 ± 6.1	2.67 ± 6.1	0 ± 6.1
c-p 5	May-18	32 ± 6.1^a^	10.33 ± 6.1^a,b^	0.67 ± 6.1^b^	0 ± 6.1^b^
**Richness**					
c-p 1	Apr-17	1 ± 0.33	1.33 ± 0.33	1.33 ± 0.33	1 ± 0.33
c-p 1	Jun-17	1.33 ± 0.33	1 ± 0.33	1.33 ± 0.33	2 ± 0.33
c-p 1	Sep-17	1.67 ± 0.33	1.33 ± 0.33	1.33 ± 0.33	1.33 ± 0.33
c-p 1	Nov-17	1.67 ± 0.33	1 ± 0.33	1.33 ± 0.33	1 ± 0.33
c-p 1	Jan-18	2 ± 0.33	2 ± 0.33	1.67 ± 0.33	1 ± 0.33
c-p 1	May-18	1.67 ± 0.33	1.33 ± 0.33	1 ± 0.33	1.33 ± 0.33
c-p 2	Apr-17	9 ± 0.87	7.67 ± 0.87	7.67 ± 0.87	7 ± 0.87
c-p 2	Jun-17	9 ± 0.87	10.67 ± 0.87	9 ± 0.87	10.67 ± 0.87
c-p 2	Sep-17	12 ± 0.87	9 ± 0.87	9.67 ± 0.87	9 ± 0.87
c-p 2	Nov-17	11.67 ± 0.87	9.67 ± 0.87	9 ± 0.87	9.67 ± 0.87
c-p 2	Jan-18	11.33 ± 0.87^a^	8.33 ± 0.87^a,b^	9.33 ± 0.87^a,b^	7.67 ± 0.87^b^
c-p 2	May-18	12 ± 0.87^a^	9 ± 0.87^a,b^	8.33 ± 0.87^b^	6.33 ± 0.87^b^
c-p 3	Apr-17	3.33 ± 0.81	2.67 ± 0.81	3.67 ± 0.81	3 ± 0.81
c-p 3	Jun-17	4.33 ± 0.81	4.33 ± 0.81	4 ± 0.81	4.33 ± 0.81
c-p 3	Sep-17	5 ± 0.81	7 ± 0.81	5 ± 0.81	5 ± 0.81
c-p 3	Nov-17	7 ± 0.81	5.33 ± 0.81	4.67 ± 0.81	4 ± 0.81
c-p 3	Jan-18	5 ± 0.81	4.33 ± 0.81	3.67 ± 0.81	3.67 ± 0.81
c-p 3	May-18	4.67 ± 0.81	5.33 ± 0.81	3.67 ± 0.81	4 ± 0.81
c-p 4	Apr-17	0.67 ± 0.51	1 ± 0.51	0.33 ± 0.51	1 ± 0.51
c-p 4	Jun-17	1.67 ± 0.51	1 ± 0.51	2.33 ± 0.51	1.67 ± 0.51
c-p 4	Sep-17	4 ± 0.51	2 ± 0.51	2.67 ± 0.51	2 ± 0.51
c-p 4	Nov-17	4.67 ± 0.51	5.67 ± 0.51	4 ± 0.51	5 ± 0.51
c-p 4	Jan-18	3 ± 0.51^a^	1 ± 0.51^a,b^	1.67 ± 0.51^a,b^	0.67 ± 0.51^b^
c-p 4	May-18	5 ± 0.51^a^	4.67 ± 0.51^a^	1 ± 0.51^b^	0 ± 0.51^b^
c-p 5	Apr-17	0 ± 0.38	0.33 ± 0.38	0 ± 0.38	0 ± 0.38
c-p 5	Jun-17	0.67 ± 0.38	0.33 ± 0.38	0.33 ± 0.38	0.67 ± 0.38
c-p 5	Sep-17	0.67 ± 0.38	0.33 ± 0.38	0.33 ± 0.38	0.67 ± 0.38
c-p 5	Nov-17	2 ± 0.38	2 ± 0.38	1.67 ± 0.38	1.33 ± 0.38
c-p 5	Jan-18	1.33 ± 0.38	0.67 ± 0.38	1 ± 0.38	0 ± 0.38
c-p 5	May-18	1.33 ± 0.38	1.33 ± 0.38	0.33 ± 0.38	0 ± 0.38

Letters indicate significant differences among treatments at each sampling time at *P* < 0.05 (Tukey–LSD test). R2M, rototill for every 2 mon; R2W, rototill for every 2 wk; SLR, surface litter removed.

**Table S4:** Nematode ecological indices in different treatments: control, SLR, R2M, and R2W at all sampling times. (mean ± pooled SE, *n* = 5).

Indices	Time	Control	SLR	R2M	R2W
λ	Apr-17	0.15 ± 0.03	0.2 ± 0.03	0.23 ± 0.03	0.2 ± 0.03
	Jun-17	0.16 ± 0.03	0.14 ± 0.03	0.12 ± 0.03	0.12 ± 0.03
	Sep-17	0.11 ± 0.03	0.14 ± 0.03	0.15 ± 0.03	0.15 ± 0.03
	Nov-17	0.1 ± 0.03	0.12 ± 0.03	0.14 ± 0.03	0.17 ± 0.03
	Jan-18	0.14 ± 0.03^a^	0.19 ± 0.03^a,b^	0.2 ± 0.03^a,b^	0.27 ± 0.03^b^
	May-18	0.11 ± 0.03^a^	0.17 ± 0.03^a,b^	0.26 ± 0.03^b^	0.24 ± 0.03^b^
H´	Apr-17	2.17 ± 0.14	1.99 ± 0.14	1.85 ± 0.14	1.89 ± 0.14
	Jun-17	2.19 ± 0.14	2.29 ± 0.14	1.83 ± 0.14	1.82 ± 0.14
	Sep-17	2.56 ± 0.14	2.28 ± 0.14	2.29 ± 0.14	2.24 ± 0.14
	Nov-17	2.65 ± 0.14	2.49 ± 0.14	2.37 ± 0.14	2.28 ± 0.14
	Jan-18	2.44 ± 0.14^a^	2.07 ± 0.14^a,b^	2.16 ± 0.14^a,b^	1.84 ± 0.14^b^
	May-18	2.63 ± 0.14^a^	2.27 ± 0.14^a,b^	1.88 ± 0.14^b^	1.82 ± 0.14^b^
NCR	Apr-17	0.72 ± 0.06	0.69 ± 0.06	0.69 ± 0.06	0.58 ± 0.06
	Jun-17	0.58 ± 0.06	0.57 ± 0.06	0.58 ± 0.06	0.55 ± 0.06
	Sep-17	0.61 ± 0.06	0.53 ± 0.06	0.56 ± 0.06	0.49 ± 0.06
	Nov-17	0.56 ± 0.06	0.57 ± 0.06	0.5 ± 0.06	0.46 ± 0.06
	Jan-18	0.43 ± 0.06	0.46 ± 0.06	0.36 ± 0.06	0.38 ± 0.06
	May-18	0.57 ± 0.06	0.64 ± 0.06	0.59 ± 0.06	0.48 ± 0.06
MI	Apr-17	1.78 ± 0.12	1.8 ± 0.14	1.65 ± 0.3	1.92 ± 0.29
	Jun-17	1.87 ± 0.28	2.02 ± 0.14	1.9 ± 0.2	1.89 ± 0.13
	Sep-17	2.01 ± 0.06	1.97 ± 0.09	2.02 ± 0.17	2.25 ± 0.13
	Nov-17	2.4 ± 0.35	2.56 ± 0.31	2.28 ± 0.12	2.31 ± 0.09
	Jan-18	2.12 ± 0.05^a^	2.1 ± 0.11^a,b^	2.05 ± 0.12^a,b^	1.92 ± 0.07^b^
	May-18	2.29 ± 0.16^a^	2.53 ± 0.28^a^	1.96 ± 0.08^b^	1.84 ± 0.14^b^
PPI	Apr-17	2.86 ± 0.08	2.88 ± 0.19	2.95 ± 0.05	2.92 ± 0.09
	Jun-17	2.87 ± 0.1	2.81 ± 0.09	2.85 ± 0.12	2.79 ± 0.18
	Sep-17	2.72 ± 0.1	2.77 ± 0.33	2.76 ± 0.05	2.8 ± 0.21
	Nov-17	2.75 ± 0.12	2.72 ± 0.08	2.61 ± 0.21	2.74 ± 0.36
	Jan-18	2.66 ± 0.24	2.81 ± 0.18	2.61 ± 0.07	2.86 ± 0.15
	May-18	2.76 ± 0.12	2.95 ± 0.04	2.96 ± 0.04	2.97 ± 0.05
BI	Apr-17	31.84 ± 3.48	28.58 ± 3.8	25.51 ± 7.97	31.75 ± 4.78
	Jun-17	26.42 ± 3.8	33.21 ± 3.33	30.42 ± 5.33	30.32 ± 5.23
	Sep-17	24.3 ± 3.48	28.84 ± 4.26	24.66 ± 3.71	29.98 ± 1.22
	Nov-17	27.25 ± 4.26	18.71 ± 1.24	24.36 ± 4.4	30.1 ± 1.72
	Jan-18	41.83 ± 1.14	39.89 ± 1.12	41.87 ± 1.95	40.39 ± 2.22
	May-18	31.21 ± 3.56	20.41 ± 3.99	35.71 ± 1.99	32.51 ± 0.24
EI	Apr-17	66.2 ± 4.03	68.84 ± 4.25	72.27 ± 9.51	60.4 ± 9.64
	Jun-17	68.42 ± 5.79	59.54 ± 2.15	64.4 ± 7.33	65.84 ± 5.59
	Sep-17	69.49 ± 3.31	65.51 ± 5.09	69.57 ± 2.54	55.23 ± 2.49
	Nov-17	52.55 ± 2.30	59.96 ± 5.13	63.86 ± 5.17	53.52 ± 3.57
	Jan-18	48.21 ± 1.01^a^	50.34 ± 3.83^a,b^	52.04 ± 1.82^a,b^	57.65 ± 2.15^b^
	May-18	52.87 ± 2.16^a^	59.36 ± 3.97^a,b^	58.99 ± 2.40^a,b^	65.12 ± 1.81^b^
SI	Apr-17	14.27 ± 1.35	22.25 ± 6.9	12.07 ± 4.79	25.55 ± 7.8
	Jun-17	31.28 ± 4.61	33.43 ± 9.58	29.57 ± 2.75	28.73 ± 4.57
	Sep-17	46.73 ± 6.6	37.35 ± 3.69	43.58 ± 11.27	51.35 ± 3.55
	Nov-17	57.72 ± 10.05	72.03 ± 3.67	58.43 ± 7.41	53.67 ± 1.65
	Jan-18	31.26 ± 2.8^a^	30.91 ± 3.68^a^	21.91 ± 6.05^a,b^	10.53 ± 2.01^b^
	May-18	51.8 ± 6.82^a^	70.23 ± 6.86^a^	25.76 ± 4.36^b^	12.89 ± 8.01^b^
CI	Apr-17	21.48 ± 4.74	22.72 ± 7.87	24.39 ± 9.93	38.52 ± 9.49
	Jun-17	31.69 ± 8.96	38.64 ± 2.75	38.6 ± 13.91	36.74 ± 12.31
	Sep-17	28.38 ± 5.15	37.83 ± 8.57	30.54 ± 2.23	59.49 ± 7.66
	Nov-17	56.33 ± 6.78	52.89 ± 10.04	45.06 ± 7.52	62.05 ± 7.36
	Jan-18	71.5 ± 3.06	65.09 ± 5.06	67.81 ± 3.87	55.13 ± 9.72
	May-18	48.61 ± 3.56	42.47 ± 8.40	37.76 ± 1.83	36.75 ± 2.08
efoot	Apr-17	169.74 ± 65	141.14 ± 39.47	260.54 ± 90.16	145.4 ± 72.04
	Jun-17	224.54 ± 68.32	88.81 ± 22.33	86.28 ± 28.13	96.08 ± 30.17
	Sep-17	206.41 ± 56.66	188.21 ± 53.38	144.31 ± 2.46	53.65 ± 16.03
	Nov-17	55 ± 13.95	75.96 ± 26.93	97.97 ± 28.81	39.16 ± 9.96
	Jan-18	49.85 ± 2.51	49.46 ± 15.85	31.49 ± 6.76	41.54 ± 6.38
	May-18	79.35 ± 16.03^a^	53.59 ± 14.81^a,b^	53.52 ± 4.22^a,b^	34.6 ± 3.36^b^
sfoot	Apr-17	9.18 ± 3.48	18.84 ± 10.55	3.97 ± 2.48	33.54 ± 14.29
	Jun-17	20.55 ± 6.99	15 ± 6.66	8.13 ± 0.89	9.7 ± 2.07
	Sep-17	31.39 ± 10.5	29.61 ± 8.07	27.88 ± 9.77	37.59 ± 11.4
	Nov-17	61.17 ± 14.29	79.08 ± 16.43	42.2 ± 10.49	31.76 ± 8.49
	Jan-18	24.22 ± 4.3^a^	8.58 ± 2.8^a,b^	6.69 ± 1.86^b^	0.9 ± 0.2^b^
	May-18	62.77 ± 21.42^a^	43.13 ± 4.79^a^	3.55 ± 0.76^b^	0.79 ± 0.48^b^

Letters indicate significant differences among treatments at each sampling time at *P* < 0.05 (Tukey–LSD test). λ, Simpson index; H´, Shannon–Weiner index. BI, basal index; CI, channel index, efoot, enrichment footprint; EI, enrichment index; MI, maturity index; NCR, nematode channel ratio; PPI, plant parasitic index; R2M, rototill for every 2 mon; R2W, rototill for every 2 wk; sfoot, structure footprint; SI, structure index; SLR, surface litter removed.

**Table S5:** Similarity percentage analysis of the nematode communities between treatments: control, surface litter removed (SLR), rototill for every two months (R2M), and rototill for every two weeks (R2W) at all sampling times.

	Av.Diss	SD	Diss/SD	Av.Abund	Av.Abund	Contrib (%)	cumsum (%)	p
**Apr-17**								
**Control vs SLR**	**Average dissimilarity = 39.35**							
*Meloidogyne*	9.66	9.12	1.06	126.33	191.33	24.54	24.54	0.195
Rhabditidae	7.98	4.82	1.66	115.67	96.00	20.29	44.83	0.952
*Acrobeloides*	4.94	2.92	1.69	46.33	53.67	12.55	57.38	0.076
*Plectus*	3.41	2.70	1.27	55.67	26.33	8.68	66.06	0.162
*Filenchus*	2.37	1.96	1.21	55.33	52.33	6.02	72.08	0.845
*Gracilacus*	1.81	1.91	0.94	11.00	14.00	4.59	76.67	0.345
*Aphelenchoides*	1.44	0.90	1.59	33.33	26.67	3.66	80.33	0.939
*Xenocriconemella*	1.36	1.01	1.34	16.00	8.00	3.44	83.77	0.805
*Pseudacrobeles*	1.18	0.75	1.58	20.00	22.00	3.00	86.77	0.593
*Tylocephalus*	0.98	0.81	1.22	16.67	4.67	2.49	89.26	0.395
Dorylaimida	0.88	1.01	0.88	3.67	7.67	2.25	91.51	0.639
*Helicotylenchus*	0.80	0.92	0.87	6.67	7.67	2.03	93.54	0.207
*Prismatolaimus*	0.64	0.52	1.24	7.00	2.67	1.63	95.17	0.091
*Acrobeles*	0.58	0.66	0.88	6.67	2.67	1.48	96.65	0.29
*Ceratoplectus*	0.45	0.33	1.38	3.00	2.67	1.14	97.79	0.362
*Teratocephalus*	0.43	0.46	0.93	3.00	1.67	1.09	98.88	0.372
*Cervidellus*	0.13	0.21	0.64	1.00	0.00	0.34	99.22	0.577
*Ecphyadophora*	0.13	0.21	0.64	1.00	0.00	0.33	99.55	0.213
*Aporcelaimellus*	0.09	0.14	0.63	0.00	0.67	0.23	99.78	0.2
*Clarkus*	0.09	0.14	0.63	0.00	0.67	0.22	100.00	0.635
*Alaimus*	0.00	0.00	NaN	0.00	0.00	0.00	100.00	1
*Boleodorus*	0.00	0.00	NaN	0.00	0.00	0.00	100.00	1
*Diphtherophora*	0.00	0.00	NaN	0.00	0.00	0.00	100.00	1
Diplogasteridae	0.00	0.00	NaN	0.00	0.00	0.00	100.00	1
*Ditylenchus*	0.00	0.00	NaN	0.00	0.00	0.00	100.00	1
*Tripyla*	0.00	0.00	NaN	0.00	0.00	0.00	100.00	1
**Control vs R2M**	**Average dissimilarity = 36.73**							
Rhabditidae	12.78	9.12	1.40	115.67	177.67	34.78	34.78	0.215
*Meloidogyne*	6.13	1.67	3.66	126.33	135.00	16.68	51.46	0.533
*Acrobeloides*	3.61	2.74	1.32	46.33	24.00	9.84	61.30	0.446
*Plectus*	2.59	0.98	2.65	55.67	29.33	7.04	68.34	0.574
*Aphelenchoides*	2.19	1.96	1.12	33.33	48.33	5.96	74.30	0.619
*Filenchus*	2.09	1.87	1.12	55.33	65.67	5.68	79.98	0.93
*Xenocriconemella*	1.17	0.95	1.24	16.00	6.33	3.19	83.17	0.872
*Tylocephalus*	0.97	0.71	1.37	16.67	9.67	2.63	85.80	0.439
*Pseudacrobeles*	0.87	0.44	1.99	20.00	11.67	2.36	88.16	0.927
*Gracilacus*	0.74	0.40	1.86	11.00	8.67	2.01	90.17	0.834
*Helicotylenchus*	0.62	0.50	1.24	6.67	2.67	1.69	91.86	0.598
*Acrobeles*	0.55	0.63	0.87	6.67	1.67	1.49	93.35	0.4
*Prismatolaimus*	0.54	0.35	1.54	7.00	2.33	1.48	94.83	0.289
*Ceratoplectus*	0.42	0.45	0.93	3.00	2.33	1.14	95.97	0.438
*Teratocephalus*	0.42	0.45	0.93	3.00	2.33	1.15	97.12	0.382
*Clarkus*	0.32	0.50	0.65	0.00	3.67	0.88	98.00	0.352
Dorylaimida	0.31	0.24	1.29	3.67	0.00	0.85	98.85	0.915
Diplogasteridae	0.15	0.24	0.65	0.00	1.67	0.42	99.27	0.298
*Cervidellus*	0.14	0.18	0.82	1.00	1.00	0.39	99.66	0.455
*Ecphyadophora*	0.12	0.19	0.65	1.00	0.00	0.34	100.00	0.279
*Alaimus*	0.00	0.00	NaN	0.00	0.00	0.00	100.00	1
*Aporcelaimellus*	0.00	0.00	NaN	0.00	0.00	0.00	100.00	1
*Boleodorus*	0.00	0.00	NaN	0.00	0.00	0.00	100.00	1
*Diphtherophora*	0.00	0.00	NaN	0.00	0.00	0.00	100.00	1
*Ditylenchus*	0.00	0.00	NaN	0.00	0.00	0.00	100.00	1
*Tripyla*	0.00	0.00	NaN	0.00	0.00	0.00	100.00	1
**Control vs R2W**	**Average dissimilarity = 42.92**							
Rhabditidae	9.32	6.32	1.47	115.67	96.67	21.72	21.72	0.822
*Filenchus*	6.44	5.87	1.10	55.33	113.67	15.01	36.73	0.205
*Meloidogyne*	5.14	3.07	1.68	126.33	104.33	11.98	48.71	0.611
*Plectus*	4.56	2.59	1.76	55.67	30.33	10.62	59.33	0.009
*Acrobeloides*	4.39	2.94	1.49	46.33	30.00	10.24	69.57	0.177
*Xenocriconemella*	2.43	1.67	1.45	16.00	29.33	5.66	75.23	0.082
*Aphelenchoides*	2.41	1.50	1.60	33.33	27.67	5.60	80.83	0.481
*Pseudacrobeles*	1.51	0.98	1.54	20.00	11.33	3.51	84.34	0.207
*Gracilacus*	1.39	1.38	1.01	11.00	12.67	3.25	87.59	0.644
Dorylaimida	1.31	0.92	1.42	3.67	14.33	3.05	90.64	0.166
*Tylocephalus*	1.30	0.86	1.51	16.67	5.00	3.02	93.66	0.044
*Acrobeles*	0.55	0.69	0.79	6.67	1.33	1.28	94.94	0.395
*Helicotylenchus*	0.55	0.69	0.79	6.67	1.33	1.27	96.21	0.723
*Prismatolaimus*	0.49	0.39	1.26	7.00	2.67	1.14	97.35	0.419
*Ceratoplectus*	0.43	0.52	0.83	3.00	1.00	1.01	98.36	0.403
*Teratocephalus*	0.41	0.64	0.64	3.00	0.00	0.95	99.31	0.385
*Cervidellus*	0.16	0.20	0.79	1.00	1.00	0.37	99.68	0.409
*Ecphyadophora*	0.14	0.21	0.64	1.00	0.00	0.32	100.00	0.141
*Alaimus*	0.00	0.00	NaN	0.00	0.00	0.00	100.00	1
*Aporcelaimellus*	0.00	0.00	NaN	0.00	0.00	0.00	100.00	1
*Boleodorus*	0.00	0.00	NaN	0.00	0.00	0.00	100.00	1
*Clarkus*	0.00	0.00	NaN	0.00	0.00	0.00	100.00	1
*Diphtherophora*	0.00	0.00	NaN	0.00	0.00	0.00	100.00	1
Diplogasteridae	0.00	0.00	NaN	0.00	0.00	0.00	100.00	1
*Ditylenchus*	0.00	0.00	NaN	0.00	0.00	0.00	100.00	1
*Tripyla*	0.00	0.00	NaN	0.00	0.00	0.00	100.00	1
**SLR vs R2M**	**Average dissimilarity = 38.89**							
Rhabditidae	13.08	7.85	1.67	96.00	177.67	33.62	33.62	0.218
*Meloidogyne*	8.80	6.86	1.28	191.33	135.00	22.64	56.26	0.366
*Acrobeloides*	2.80	2.10	1.33	53.67	24.00	7.20	63.46	0.723
*Aphelenchoides*	2.62	2.19	1.19	26.67	48.33	6.74	70.20	0.356
*Filenchus*	2.33	1.67	1.39	52.33	65.67	5.99	76.19	0.871
*Gracilacus*	1.74	1.64	1.06	14.00	8.67	4.47	80.66	0.406
*Plectus*	1.40	1.19	1.18	26.33	29.33	3.60	84.26	0.986
*Pseudacrobeles*	1.21	0.65	1.87	22.00	11.67	3.12	87.38	0.553
Dorylaimida	0.84	1.11	0.76	7.67	0.00	2.17	89.55	0.682
*Xenocriconemella*	0.73	0.61	1.20	8.00	6.33	1.87	91.42	0.962
*Helicotylenchus*	0.70	0.62	1.14	7.67	2.67	1.81	93.23	0.395
*Tylocephalus*	0.62	0.29	2.11	4.67	9.67	1.59	94.82	0.811
*Clarkus*	0.36	0.43	0.84	0.67	3.67	0.93	95.75	0.244
*Prismatolaimus*	0.36	0.44	0.81	2.67	2.33	0.92	96.67	0.752
*Ceratoplectus*	0.34	0.23	1.51	2.67	2.33	0.88	97.55	0.634
*Teratocephalus*	0.32	0.35	0.94	1.67	2.33	0.83	98.38	0.609
*Acrobeles*	0.27	0.28	0.95	2.67	1.67	0.69	99.07	0.7
Diplogasteridae	0.15	0.24	0.65	0.00	1.67	0.39	99.46	0.298
*Cervidellus*	0.13	0.20	0.64	0.00	1.00	0.33	99.79	0.611
*Aporcelaimellus*	0.08	0.13	0.65	0.67	0.00	0.21	100.00	0.286
*Alaimus*	0.00	0.00	NaN	0.00	0.00	0.00	100.00	1
*Boleodorus*	0.00	0.00	NaN	0.00	0.00	0.00	100.00	1
*Diphtherophora*	0.00	0.00	NaN	0.00	0.00	0.00	100.00	1
*Ditylenchus*	0.00	0.00	NaN	0.00	0.00	0.00	100.00	1
*Ecphyadophora*	0.00	0.00	NaN	0.00	0.00	0.00	100.00	1
*Tripyla*	0.00	0.00	NaN	0.00	0.00	0.00	100.00	1
**SLR vs R2W**	**Average dissimilarity = 42.38**							
Rhabditidae	9.05	5.14	1.76	96.00	96.67	21.36	21.36	0.859
*Meloidogyne*	8.47	9.36	0.91	191.33	104.33	20.00	41.36	0.416
*Filenchus*	6.78	5.84	1.16	52.33	113.67	16.00	57.36	0.144
*Acrobeloides*	3.02	2.28	1.33	53.67	30.00	7.13	64.49	0.67
*Plectus*	2.67	1.92	1.39	26.33	30.33	6.30	70.79	0.552
*Xenocriconemella*	2.37	1.45	1.64	8.00	29.33	5.61	76.40	0.133
*Aphelenchoides*	2.32	1.20	1.93	26.67	27.67	5.47	81.87	0.547
*Gracilacus*	1.99	1.85	1.07	14.00	12.67	4.69	86.56	0.231
*Pseudacrobeles*	1.64	1.14	1.44	22.00	11.33	3.88	90.44	0.09
Dorylaimida	1.32	1.00	1.32	7.67	14.33	3.13	93.57	0.175
*Helicotylenchus*	0.64	0.83	0.77	7.67	1.33	1.50	95.07	0.545
*Tylocephalus*	0.62	0.37	1.68	4.67	5.00	1.46	96.53	0.814
*Prismatolaimus*	0.39	0.27	1.46	2.67	2.67	0.93	97.46	0.648
*Ceratoplectus*	0.28	0.26	1.06	2.67	1.00	0.65	98.11	0.783
*Acrobeles*	0.25	0.28	0.90	2.67	1.33	0.60	98.71	0.697
*Teratocephalus*	0.23	0.36	0.64	1.67	0.00	0.55	99.26	0.77
*Cervidellus*	0.13	0.21	0.64	0.00	1.00	0.30	99.56	0.592
*Aporcelaimellus*	0.09	0.14	0.64	0.67	0.00	0.22	99.78	0.144
*Clarkus*	0.09	0.14	0.64	0.67	0.00	0.22	100.00	0.585
*Alaimus*	0.00	0.00	NaN	0.00	0.00	0.00	100.00	1
*Boleodorus*	0.00	0.00	NaN	0.00	0.00	0.00	100.00	1
*Diphtherophora*	0.00	0.00	NaN	0.00	0.00	0.00	100.00	1
Diplogasteridae	0.00	0.00	NaN	0.00	0.00	0.00	100.00	1
*Ditylenchus*	0.00	0.00	NaN	0.00	0.00	0.00	100.00	1
*Ecphyadophora*	0.00	0.00	NaN	0.00	0.00	0.00	100.00	1
*Tripyla*	0.00	0.00	NaN	0.00	0.00	0.00	100.00	1
**R2M vs R2W**	**Average dissimilarity = 39.39**							
Rhabditidae	13.96	10.26	1.36	177.67	96.67	35.44	35.44	0.096
*Filenchus*	5.99	5.23	1.15	65.67	113.67	15.21	50.65	0.296
*Aphelenchoides*	3.29	2.56	1.29	48.33	27.67	8.36	59.01	0.116
*Meloidogyne*	2.97	1.58	1.88	135.00	104.33	7.53	66.54	0.929
*Plectus*	2.94	0.71	4.15	29.33	30.33	7.45	73.99	0.381
*Xenocriconemella*	2.26	1.66	1.36	6.33	29.33	5.73	79.72	0.152
*Gracilacus*	1.36	1.16	1.17	8.67	12.67	3.46	83.18	0.69
Dorylaimida	1.34	1.04	1.29	0.00	14.33	3.39	86.57	0.161
*Acrobeloides*	1.32	1.48	0.89	24.00	30.00	3.35	89.92	0.969
*Pseudacrobeles*	1.12	0.61	1.82	11.67	11.33	2.83	92.75	0.701
*Tylocephalus*	0.77	0.55	1.39	9.67	5.00	1.96	94.71	0.592
*Prismatolaimus*	0.36	0.24	1.50	2.33	2.67	0.90	95.61	0.755
*Clarkus*	0.33	0.51	0.65	3.67	0.00	0.85	96.46	0.315
*Ceratoplectus*	0.32	0.37	0.87	2.33	1.00	0.82	97.28	0.66
*Teratocephalus*	0.30	0.47	0.64	2.33	0.00	0.77	98.05	0.623
*Helicotylenchus*	0.27	0.23	1.16	2.67	1.33	0.68	98.73	0.816
*Acrobeles*	0.19	0.22	0.86	1.67	1.33	0.49	99.22	0.822
Diplogasteridae	0.16	0.24	0.65	1.67	0.00	0.40	99.62	0.212
*Cervidellus*	0.15	0.18	0.82	1.00	1.00	0.38	100.00	0.419
*Alaimus*	0.00	0.00	NaN	0.00	0.00	0.00	100.00	1
*Aporcelaimellus*	0.00	0.00	NaN	0.00	0.00	0.00	100.00	1
*Boleodorus*	0.00	0.00	NaN	0.00	0.00	0.00	100.00	1
*Diphtherophora*	0.00	0.00	NaN	0.00	0.00	0.00	100.00	1
*Ditylenchus*	0.00	0.00	NaN	0.00	0.00	0.00	100.00	1
*Ecphyadophora*	0.00	0.00	NaN	0.00	0.00	0.00	100.00	1
*Tripyla*	0.00	0.00	NaN	0.00	0.00	0.00	100.00	1
**Jun-17**								
**Control vs SLR**	**Average dissimilarity = 39.91**							
Rhabditidae	7.60	6.68	1.14	147.67	57.00	19.05	19.05	0.418
*Filenchus*	6.40	4.25	1.51	158.67	111.67	16.03	35.08	0.448
*Meloidogyne*	5.97	5.02	1.19	165.33	104.33	14.95	50.03	0.593
*Plectus*	2.53	1.37	1.84	72.33	40.00	6.34	56.37	0.74
*Xenocriconemella*	2.05	1.53	1.34	29.00	30.67	5.14	61.51	0.851
*Prismatolaimus*	1.84	1.48	1.25	19.00	28.00	4.61	66.12	0.503
*Aphelenchoides*	1.72	1.04	1.66	29.33	20.33	4.32	70.44	0.311
*Acrobeloides*	1.44	0.93	1.54	18.67	27.00	3.60	74.04	0.813
*Helicotylenchus*	1.39	1.21	1.16	20.67	8.67	3.50	77.54	0.472
*Diphtherophora*	1.28	1.22	1.05	13.33	9.00	3.21	80.75	0.425
*Ditylenchus*	1.24	0.85	1.46	16.67	2.33	3.10	83.85	0.085
*Pseudacrobeles*	1.10	0.76	1.43	18.67	5.67	2.75	86.60	0.285
*Boleodorus*	0.98	0.94	1.04	10.00	10.67	2.44	89.04	0.776
*Alaimus*	0.81	0.71	1.14	10.33	5.33	2.04	91.08	0.515
*Gracilacus*	0.73	0.80	0.91	19.67	13.67	1.82	92.90	0.918
*Ceratoplectus*	0.71	0.64	1.10	5.00	12.33	1.77	94.67	0.638
*Tylocephalus*	0.40	0.29	1.39	9.67	7.67	1.00	95.67	0.833
*Acrobeles*	0.39	0.20	1.92	1.67	6.33	0.97	96.64	0.91
*Aporcelaimellus*	0.37	0.32	1.16	4.67	2.33	0.93	97.57	0.418
*Clarkus*	0.27	0.18	1.45	3.00	2.33	0.66	98.23	0.357
*Ecphyadophora*	0.25	0.21	1.20	0.00	3.33	0.64	98.87	0.376
*Tripyla*	0.16	0.19	0.87	1.67	1.33	0.41	99.28	0.622
*Teratocephalus*	0.15	0.23	0.66	0.00	1.67	0.39	99.67	0.503
Diplogasteridae	0.13	0.20	0.66	1.67	0.00	0.33	100.00	0.832
*Cervidellus*	0.00	0.00	NaN	0.00	0.00	0.00	100.00	1
Dorylaimida	0.00	0.00	NaN	0.00	0.00	0.00	100.00	1
**Control vs R2M**	**Average dissimilarity = 53.30**							
*Meloidogyne*	10.70	4.70	2.28	165.33	46.33	20.07	20.07	0.017
Rhabditidae	9.06	7.57	1.20	147.67	56.33	17.00	37.07	0.191
*Filenchus*	8.88	6.15	1.44	158.67	75.67	16.66	53.73	0.046
*Plectus*	5.19	1.10	4.70	72.33	16.33	9.74	63.47	0.007
*Aphelenchoides*	2.27	1.31	1.73	29.33	12.00	4.26	67.73	0.014
*Xenocriconemella*	2.18	1.34	1.63	29.00	9.00	4.09	71.82	0.796
*Helicotylenchus*	1.76	1.21	1.45	20.67	2.33	3.30	75.12	0.119
*Gracilacus*	1.66	1.11	1.50	19.67	2.00	3.12	78.24	0.057
*Acrobeloides*	1.53	1.02	1.50	18.67	28.33	2.88	81.12	0.697
*Ditylenchus*	1.26	0.89	1.42	16.67	5.00	2.37	83.49	0.08
*Diphtherophora*	1.26	1.90	0.67	13.33	0.00	2.37	85.86	0.433
*Prismatolaimus*	1.22	0.65	1.89	19.00	6.00	2.28	88.14	0.643
*Pseudacrobeles*	1.21	0.85	1.43	18.67	6.00	2.28	90.42	0.17
*Boleodorus*	1.02	0.96	1.07	10.00	7.33	1.92	92.34	0.74
*Tylocephalus*	0.81	0.38	2.11	9.67	0.67	1.52	93.86	0.123
*Alaimus*	0.79	0.60	1.33	10.33	6.33	1.49	95.35	0.544
*Ceratoplectus*	0.57	0.44	1.31	5.00	7.33	1.07	96.42	0.778
*Acrobeles*	0.56	0.65	0.87	1.67	5.67	1.06	97.48	0.79
*Aporcelaimellus*	0.40	0.37	1.08	4.67	1.33	0.76	98.24	0.281
*Clarkus*	0.27	0.18	1.47	3.00	0.67	0.50	98.74	0.356
Diplogasteridae	0.18	0.19	0.92	1.67	0.67	0.34	99.08	0.709
*Tripyla*	0.18	0.19	0.92	1.67	0.67	0.33	99.41	0.535
*Ecphyadophora*	0.13	0.10	1.31	0.00	1.33	0.24	99.65	0.841
*Teratocephalus*	0.07	0.10	0.66	0.00	0.67	0.12	99.77	0.79
*Cervidellus*	0.06	0.09	0.66	0.00	0.67	0.12	99.89	0.745
Dorylaimida	0.06	0.09	0.66	0.00	0.67	0.11	100.00	0.78
**Control vs R2W**	**Average dissimilarity = 49.23**							
*Meloidogyne*	10.62	4.58	2.32	165.33	40.33	21.56	21.56	0.012
Rhabditidae	8.27	7.16	1.16	147.67	61.67	16.79	38.35	0.326
*Filenchus*	7.69	5.45	1.41	158.67	94.67	15.62	53.97	0.152
*Plectus*	4.56	1.56	2.92	72.33	20.33	9.26	63.23	0.014
*Xenocriconemella*	3.30	2.58	1.28	29.00	40.67	6.71	69.94	0.544
*Aphelenchoides*	2.16	1.33	1.62	29.33	11.33	4.39	74.33	0.034
*Helicotylenchus*	1.55	1.24	1.25	20.67	7.33	3.14	77.47	0.325
*Diphtherophora*	1.33	1.42	0.93	13.33	5.00	2.70	80.17	0.369
*Acrobeloides*	1.28	0.77	1.66	18.67	19.00	2.59	82.76	0.899
*Ditylenchus*	1.17	0.76	1.54	16.67	5.00	2.37	85.13	0.141
*Pseudacrobeles*	1.14	0.64	1.79	18.67	6.67	2.32	87.45	0.211
*Gracilacus*	1.04	0.89	1.17	19.67	12.67	2.12	89.57	0.642
*Alaimus*	0.97	0.89	1.10	10.33	0.00	1.98	91.55	0.226
*Boleodorus*	0.90	0.77	1.16	10.00	6.67	1.82	93.37	0.835
*Prismatolaimus*	0.69	0.49	1.40	19.00	12.33	1.39	94.76	0.981
*Acrobeles*	0.60	0.56	1.08	1.67	7.67	1.22	95.98	0.781
*Ceratoplectus*	0.40	0.35	1.17	5.00	5.33	0.82	96.80	0.965
*Aporcelaimellus*	0.38	0.31	1.20	4.67	1.33	0.77	97.57	0.406
*Tylocephalus*	0.35	0.32	1.10	9.67	6.33	0.71	98.28	0.912
*Clarkus*	0.28	0.21	1.32	3.00	0.00	0.56	98.84	0.318
Diplogasteridae	0.22	0.19	1.16	1.67	2.67	0.45	99.29	0.483
*Tripyla*	0.17	0.18	0.93	1.67	0.67	0.34	99.63	0.589
*Cervidellus*	0.06	0.10	0.66	0.00	0.67	0.13	99.76	0.753
Dorylaimida	0.06	0.10	0.66	0.00	0.67	0.13	99.89	0.761
*Ecphyadophora*	0.06	0.08	0.66	0.00	0.67	0.11	100.00	0.995
*Teratocephalus*	0.00	0.00	NaN	0.00	0.00	0.00	100.00	1
**SLR vs R2M**	**Average dissimilarity = 40.41**							
*Meloidogyne*	6.59	5.33	1.24	104.33	46.33	16.30	16.30	0.5
Rhabditidae	4.87	3.00	1.62	57.00	56.33	12.05	28.35	0.798
*Filenchus*	4.78	3.85	1.24	111.67	75.67	11.83	40.18	0.8
*Xenocriconemella*	3.15	2.66	1.18	30.67	9.00	7.79	47.97	0.559
*Prismatolaimus*	3.09	3.08	1.00	28.00	6.00	7.65	55.62	0.048
*Plectus*	3.00	1.70	1.77	40.00	16.33	7.43	63.05	0.447
*Acrobeloides*	2.07	1.74	1.19	27.00	28.33	5.14	68.19	0.266
*Gracilacus*	1.42	0.22	6.62	13.67	2.00	3.52	71.71	0.192
*Boleodorus*	1.42	1.35	1.05	10.67	7.33	3.52	75.23	0.307
*Ceratoplectus*	1.11	1.01	1.10	12.33	7.33	2.73	77.96	0.139
*Diphtherophora*	1.03	1.00	1.03	9.00	0.00	2.55	80.51	0.527
*Helicotylenchus*	1.03	1.10	0.93	8.67	2.33	2.55	83.06	0.815
*Aphelenchoides*	1.02	0.80	1.27	20.33	12.00	2.51	85.57	0.789
*Tylocephalus*	1.00	0.79	1.26	7.67	0.67	2.47	88.04	0.02
*Acrobeles*	0.96	0.48	1.99	6.33	5.67	2.38	90.42	0.232
*Alaimus*	0.95	0.46	2.08	5.33	6.33	2.36	92.78	0.267
*Pseudacrobeles*	0.65	0.57	1.15	5.67	6.00	1.61	94.39	0.905
*Ditylenchus*	0.60	0.55	1.08	2.33	5.00	1.48	95.87	0.857
*Ecphyadophora*	0.34	0.23	1.46	3.33	1.33	0.84	96.71	0.048
*Aporcelaimellus*	0.32	0.33	0.97	2.33	1.33	0.79	97.50	0.595
*Teratocephalus*	0.29	0.34	0.85	1.67	0.67	0.71	98.21	0.082
*Clarkus*	0.28	0.31	0.93	2.33	0.67	0.71	98.92	0.311
*Tripyla*	0.18	0.18	0.99	1.33	0.67	0.44	99.36	0.516
Diplogasteridae	0.09	0.14	0.64	0.00	0.67	0.23	99.59	0.952
*Cervidellus*	0.09	0.13	0.65	0.00	0.67	0.21	99.80	0.53
Dorylaimida	0.08	0.12	0.65	0.00	0.67	0.20	100.00	0.611
**SLR vs R2W**	**Average dissimilarity = 39.48**							
*Meloidogyne*	6.83	5.03	1.36	104.33	40.33	17.31	17.31	0.435
*Filenchus*	5.12	3.61	1.42	111.67	94.67	12.97	30.28	0.759
Rhabditidae	4.60	3.00	1.54	57.00	61.67	11.65	41.93	0.84
*Xenocriconemella*	4.55	4.09	1.11	30.67	40.67	11.52	53.45	0.264
*Prismatolaimus*	2.81	2.43	1.16	28.00	12.33	7.12	60.57	0.132
*Plectus*	2.61	1.70	1.54	40.00	20.33	6.62	67.19	0.716
*Acrobeloides*	1.83	1.36	1.35	27.00	19.00	4.65	71.84	0.474
*Boleodorus*	1.28	1.04	1.23	10.67	6.67	3.24	75.08	0.479
*Helicotylenchus*	1.19	1.34	0.89	8.67	7.33	3.00	78.08	0.645
*Aphelenchoides*	1.04	0.84	1.23	20.33	11.33	2.63	80.71	0.738
*Ceratoplectus*	1.03	1.01	1.03	12.33	5.33	2.61	83.32	0.233
*Diphtherophora*	0.90	0.73	1.24	9.00	5.00	2.28	85.60	0.611
*Gracilacus*	0.83	0.48	1.72	13.67	12.67	2.10	87.70	0.858
*Pseudacrobeles*	0.78	0.70	1.10	5.67	6.67	1.96	89.66	0.779
*Acrobeles*	0.75	0.52	1.44	6.33	7.67	1.90	91.56	0.598
*Ditylenchus*	0.58	0.50	1.15	2.33	5.00	1.47	93.03	0.887
*Alaimus*	0.58	0.87	0.66	5.33	0.00	1.47	94.50	0.843
*Tylocephalus*	0.42	0.45	0.92	7.67	6.33	1.06	95.56	0.785
*Ecphyadophora*	0.35	0.27	1.27	3.33	0.67	0.87	96.43	0.035
*Aporcelaimellus*	0.30	0.22	1.33	2.33	1.33	0.76	97.19	0.679
Diplogasteridae	0.30	0.25	1.20	0.00	2.67	0.74	97.93	0.143
*Teratocephalus*	0.25	0.37	0.66	1.67	0.00	0.63	98.56	0.191
*Clarkus*	0.22	0.34	0.66	2.33	0.00	0.57	99.13	0.566
*Tripyla*	0.17	0.17	1.00	1.33	0.67	0.43	99.56	0.589
*Cervidellus*	0.09	0.13	0.65	0.00	0.67	0.22	99.78	0.517
Dorylaimida	0.09	0.13	0.65	0.00	0.67	0.22	100.00	0.45
**R2M vs R2W**	**Average dissimilarity = 35.56**							
Rhabditidae	6.37	4.68	1.36	56.33	61.67	17.91	17.91	0.588
*Xenocriconemella*	5.57	5.65	0.99	9.00	40.67	15.67	33.58	0.066
*Filenchus*	4.67	4.63	1.01	75.67	94.67	13.13	46.71	0.842
*Acrobeloides*	2.17	1.69	1.29	28.33	19.00	6.11	52.82	0.177
*Gracilacus*	1.60	1.09	1.47	2.00	12.67	4.50	57.32	0.077
*Plectus*	1.53	1.16	1.31	16.33	20.33	4.29	61.61	0.996
*Meloidogyne*	1.42	1.42	1.00	46.33	40.33	4.01	65.62	0.995
*Prismatolaimus*	1.42	1.09	1.31	6.00	12.33	3.99	69.61	0.552
*Boleodorus*	1.34	1.13	1.19	7.33	6.67	3.77	73.38	0.405
*Acrobeles*	1.20	1.09	1.10	5.67	7.67	3.36	76.74	0.063
*Helicotylenchus*	1.13	1.21	0.94	2.33	7.33	3.18	79.92	0.733
*Pseudacrobeles*	1.01	0.73	1.39	6.00	6.67	2.83	82.75	0.446
*Alaimus*	0.98	0.51	1.93	6.33	0.00	2.75	85.50	0.239
*Ceratoplectus*	0.89	0.62	1.42	7.33	5.33	2.50	88.00	0.382
*Tylocephalus*	0.85	0.12	6.83	0.67	6.33	2.37	90.37	0.077
*Diphtherophora*	0.72	0.64	1.12	0.00	5.00	2.02	92.39	0.732
*Ditylenchus*	0.69	0.57	1.20	5.00	5.00	1.93	94.32	0.803
*Aphelenchoides*	0.59	0.52	1.15	12.00	11.33	1.67	95.99	0.972
Diplogasteridae	0.35	0.25	1.41	0.67	2.67	0.98	96.97	0.035
*Aporcelaimellus*	0.27	0.18	1.48	1.33	1.33	0.76	97.73	0.771
*Ecphyadophora*	0.17	0.17	1.04	1.33	0.67	0.48	98.21	0.651
*Tripyla*	0.14	0.17	0.84	0.67	0.67	0.40	98.61	0.689
*Cervidellus*	0.14	0.17	0.83	0.67	0.67	0.39	99.00	0.073
Dorylaimida	0.14	0.17	0.82	0.67	0.67	0.39	99.39	0.09
*Clarkus*	0.11	0.17	0.66	0.67	0.00	0.30	99.69	0.934
*Teratocephalus*	0.11	0.17	0.66	0.67	0.00	0.31	100.00	0.516
**Sep-17**								
**Control vs SLR**	**Average dissimilarity = 43.89**							
*Filenchus*	5.62	3.43	1.64	116.33	196.33	12.80	12.80	0.039
Rhabditidae	5.48	3.60	1.52	134.67	120.00	12.49	25.29	0.648
*Helicotylenchus*	4.72	6.18	0.76	6.67	74.67	10.74	36.03	0.348
*Gracilacus*	4.39	5.11	0.86	24.00	77.00	10.00	46.03	0.477
*Meloidogyne*	3.42	3.53	0.97	66.67	97.00	7.79	53.82	0.622
*Plectus*	2.89	2.40	1.20	50.00	63.00	6.58	60.40	0.436
*Xenocriconemella*	2.78	1.86	1.49	63.33	41.67	6.32	66.72	0.878
*Aphelenchoides*	2.14	1.66	1.29	36.00	48.00	4.87	71.59	0.427
*Prismatolaimus*	1.94	1.46	1.34	28.33	37.67	4.43	76.02	0.694
*Acrobeloides*	1.51	1.11	1.36	11.67	31.33	3.43	79.45	0.814
*Alaimus*	1.49	1.17	1.27	33.67	13.00	3.39	82.84	0.486
*Ditylenchus*	1.34	0.60	2.22	24.67	4.33	3.05	85.89	0.182
*Pseudacrobeles*	0.80	0.54	1.48	19.33	8.00	1.81	87.70	0.479
*Boleodorus*	0.79	0.16	4.97	13.33	1.00	1.80	89.50	0.153
*Teratocephalus*	0.71	0.62	1.14	12.33	7.00	1.60	91.10	0.509
*Acrobeles*	0.69	0.47	1.48	7.67	12.33	1.58	92.68	0.742
*Ceratoplectus*	0.57	0.42	1.37	9.33	5.33	1.30	93.98	0.256
*Diphtherophora*	0.41	0.16	2.49	1.67	8.00	0.93	94.91	0.245
Dorylaimida	0.39	0.34	1.13	3.00	5.33	0.89	95.80	0.908
*Ecphyadophora*	0.38	0.31	1.23	5.33	0.00	0.87	96.67	0.101
*Cervidellus*	0.29	0.30	0.95	2.33	3.33	0.65	97.32	0.438
Diplogasteridae	0.28	0.23	1.22	2.67	3.67	0.63	97.95	0.192
*Tylocephalus*	0.27	0.21	1.30	5.67	3.33	0.62	98.57	0.531
*Tripyla*	0.24	0.24	0.99	3.67	2.33	0.54	99.11	0.322
*Aporcelaimellus*	0.23	0.22	1.06	3.00	2.33	0.52	99.63	0.78
*Clarkus*	0.16	0.14	1.17	2.67	1.67	0.37	100.00	0.745
**Control vs R2M**	**Average dissimilarity = 38.78**							
Rhabditidae	6.47	1.38	4.70	134.67	93.67	16.68	16.68	0.314
*Xenocriconemella*	4.56	3.29	1.39	63.33	16.00	11.77	28.45	0.456
*Meloidogyne*	3.63	2.71	1.34	66.67	76.00	9.35	37.80	0.553
*Filenchus*	3.31	2.50	1.32	116.33	133.00	8.52	46.32	0.613
*Plectus*	2.98	1.77	1.69	50.00	10.67	7.69	54.01	0.385
*Acrobeloides*	2.19	2.03	1.08	11.67	36.33	5.64	59.65	0.41
*Prismatolaimus*	2.13	1.48	1.44	28.33	20.67	5.49	65.14	0.543
*Alaimus*	2.00	1.58	1.26	33.67	20.00	5.17	70.31	0.106
*Aphelenchoides*	1.76	0.98	1.80	36.00	18.00	4.54	74.85	0.514
*Acrobeles*	1.08	0.88	1.23	7.67	12.33	2.80	77.65	0.364
*Ditylenchus*	0.99	0.92	1.08	24.67	15.33	2.56	80.21	0.674
*Pseudacrobeles*	0.99	0.77	1.28	19.33	8.67	2.54	82.75	0.15
*Gracilacus*	0.91	0.74	1.23	24.00	17.33	2.34	85.09	0.963
*Teratocephalus*	0.88	0.81	1.08	12.33	2.33	2.27	87.36	0.109
*Helicotylenchus*	0.78	1.09	0.71	6.67	1.00	2.00	89.36	0.645
*Boleodorus*	0.60	0.45	1.32	13.33	10.33	1.55	90.91	0.656
*Ceratoplectus*	0.51	0.35	1.45	9.33	5.00	1.31	92.22	0.511
*Ecphyadophora*	0.50	0.41	1.24	5.33	0.00	1.30	93.52	0.017
Dorylaimida	0.48	0.48	1.02	3.00	4.33	1.25	94.77	0.812
*Aporcelaimellus*	0.40	0.35	1.15	3.00	3.67	1.03	95.80	0.316
*Tylocephalus*	0.38	0.28	1.36	5.67	1.33	0.98	96.78	0.089
*Clarkus*	0.30	0.32	0.92	2.67	4.00	0.76	97.54	0.364
*Cervidellus*	0.28	0.35	0.79	2.33	1.00	0.72	98.26	0.485
*Tripyla*	0.27	0.22	1.21	3.67	0.00	0.70	98.96	0.197
*Diphtherophora*	0.21	0.25	0.84	1.67	1.33	0.53	99.49	0.962
Diplogasteridae	0.20	0.15	1.32	2.67	0.00	0.51	100.00	0.556
**Control vs R2W**	**Average dissimilarity = 44.55**							
Rhabditidae	8.50	5.02	1.69	134.67	30.00	19.08	19.08	0.035
*Xenocriconemella*	5.12	3.74	1.37	63.33	92.00	11.49	30.57	0.376
*Meloidogyne*	3.48	2.55	1.36	66.67	33.67	7.82	38.39	0.604
*Filenchus*	3.06	1.87	1.63	116.33	123.33	6.87	45.26	0.719
*Prismatolaimus*	2.86	2.40	1.19	28.33	41.67	6.41	51.67	0.119
*Gracilacus*	2.85	1.81	1.57	24.00	28.33	6.41	58.08	0.635
*Plectus*	2.52	1.80	1.41	50.00	17.67	5.66	63.74	0.526
*Acrobeloides*	2.43	2.26	1.08	11.67	37.67	5.45	69.19	0.248
*Aphelenchoides*	1.88	1.05	1.79	36.00	16.67	4.21	73.40	0.504
*Alaimus*	1.85	1.25	1.48	33.67	16.00	4.15	77.55	0.188
*Pseudacrobeles*	1.65	0.48	3.46	19.33	1.00	3.70	81.25	0.004
*Ditylenchus*	1.48	0.76	1.95	24.67	13.33	3.31	84.56	0.089
*Teratocephalus*	0.90	0.84	1.07	12.33	2.00	2.03	86.59	0.077
Dorylaimida	0.84	0.65	1.30	3.00	10.67	1.89	88.48	0.193
*Helicotylenchus*	0.81	1.01	0.80	6.67	2.00	1.81	90.29	0.622
*Ceratoplectus*	0.66	0.48	1.37	9.33	1.00	1.49	91.78	0.102
*Boleodorus*	0.63	0.46	1.37	13.33	6.00	1.42	93.20	0.511
*Acrobeles*	0.59	0.47	1.26	7.67	2.00	1.33	94.53	0.833
*Ecphyadophora*	0.52	0.42	1.24	5.33	0.00	1.16	95.69	0.012
*Tylocephalus*	0.39	0.28	1.37	5.67	1.00	0.87	96.56	0.053
*Diphtherophora*	0.32	0.25	1.26	1.67	3.67	0.72	97.28	0.719
*Cervidellus*	0.31	0.12	2.46	2.33	3.00	0.69	97.97	0.307
*Tripyla*	0.28	0.23	1.21	3.67	0.00	0.62	98.59	0.182
*Aporcelaimellus*	0.23	0.15	1.53	3.00	2.00	0.51	99.10	0.773
*Clarkus*	0.20	0.15	1.33	2.67	0.00	0.45	99.55	0.604
Diplogasteridae	0.20	0.15	1.33	2.67	0.00	0.45	100.00	0.553
**SLR vs R2M**	**Average dissimilarity = 40.47**							
*Helicotylenchus*	5.18	7.14	0.73	74.67	1.00	12.80	12.80	0.154
*Gracilacus*	4.84	5.83	0.83	77.00	17.33	11.95	24.75	0.315
*Filenchus*	4.51	2.83	1.60	196.33	133.00	11.15	35.90	0.241
Rhabditidae	3.61	3.10	1.17	120.00	93.67	8.93	44.83	0.941
*Plectus*	3.57	3.34	1.07	63.00	10.67	8.82	53.65	0.114
*Meloidogyne*	2.92	2.61	1.12	97.00	76.00	7.20	60.85	0.796
*Xenocriconemella*	2.62	2.34	1.12	41.67	16.00	6.47	67.32	0.903
*Aphelenchoides*	2.16	2.38	0.91	48.00	18.00	5.35	72.67	0.424
*Acrobeloides*	1.71	1.09	1.57	31.33	36.33	4.23	76.90	0.755
*Prismatolaimus*	1.58	1.25	1.27	37.67	20.67	3.91	80.81	0.904
*Alaimus*	1.09	1.18	0.93	13.00	20.00	2.69	83.50	0.768
*Acrobeles*	1.05	0.80	1.32	12.33	12.33	2.61	86.11	0.418
*Ditylenchus*	0.90	0.60	1.50	4.33	15.33	2.22	88.33	0.822
*Boleodorus*	0.73	0.50	1.47	1.00	10.33	1.81	90.14	0.249
*Pseudacrobeles*	0.55	0.36	1.51	8.00	8.67	1.35	91.49	0.922
*Ceratoplectus*	0.51	0.21	2.45	5.33	5.00	1.26	92.75	0.516
Dorylaimida	0.49	0.55	0.89	5.33	4.33	1.22	93.97	0.816
*Diphtherophora*	0.47	0.23	2.04	8.00	1.33	1.18	95.15	0.083
*Teratocephalus*	0.43	0.20	2.15	7.00	2.33	1.06	96.21	0.557
*Aporcelaimellus*	0.34	0.34	1.00	2.33	3.67	0.85	97.06	0.482
*Clarkus*	0.29	0.27	1.08	1.67	4.00	0.71	97.77	0.403
*Cervidellus*	0.26	0.30	0.85	3.33	1.00	0.63	98.40	0.649
Diplogasteridae	0.23	0.35	0.67	3.67	0.00	0.57	98.97	0.381
*Tripyla*	0.21	0.32	0.66	2.33	0.00	0.53	99.50	0.443
*Tylocephalus*	0.20	0.16	1.23	3.33	1.33	0.50	100.00	0.698
*Ecphyadophora*	0.00	0.00	NaN	0.00	0.00	0.00	100.00	1
**SLR vs R2W**	**Average dissimilarity = 47.26**							
Rhabditidae	6.43	4.63	1.39	120.00	30.00	13.60	13.60	0.278
*Gracilacus*	5.58	5.42	1.03	77.00	28.33	11.81	25.41	0.142
*Helicotylenchus*	5.23	7.21	0.73	74.67	2.00	11.07	36.48	0.126
*Meloidogyne*	5.09	3.58	1.42	97.00	33.67	10.76	47.24	0.098
*Filenchus*	5.06	2.75	1.84	196.33	123.33	10.70	57.94	0.083
*Xenocriconemella*	4.26	3.71	1.15	41.67	92.00	9.01	66.95	0.593
*Plectus*	3.23	3.32	0.97	63.00	17.67	6.83	73.78	0.26
*Aphelenchoides*	2.36	2.33	1.01	48.00	16.67	5.00	78.78	0.281
*Prismatolaimus*	1.82	1.44	1.26	37.67	41.67	3.85	82.63	0.724
*Acrobeloides*	1.80	1.21	1.49	31.33	37.67	3.81	86.44	0.747
*Acrobeles*	0.83	0.67	1.24	12.33	2.00	1.75	88.19	0.582
*Ditylenchus*	0.82	0.87	0.95	4.33	13.33	1.74	89.93	0.909
Dorylaimida	0.75	0.64	1.18	5.33	10.67	1.59	91.52	0.426
*Pseudacrobeles*	0.50	0.43	1.15	8.00	1.00	1.06	92.58	0.972
*Alaimus*	0.50	0.45	1.11	13.00	16.00	1.05	93.63	0.972
*Teratocephalus*	0.44	0.21	2.08	7.00	2.00	0.93	94.56	0.533
*Boleodorus*	0.43	0.31	1.39	1.00	6.00	0.91	95.47	0.969
*Ceratoplectus*	0.40	0.50	0.81	5.33	1.00	0.85	96.32	0.763
*Diphtherophora*	0.39	0.25	1.57	8.00	3.67	0.82	97.14	0.325
*Cervidellus*	0.32	0.13	2.42	3.33	3.00	0.68	97.82	0.258
*Aporcelaimellus*	0.25	0.21	1.19	2.33	2.00	0.53	98.35	0.694
Diplogasteridae	0.24	0.35	0.67	3.67	0.00	0.49	98.84	0.387
*Tripyla*	0.22	0.33	0.67	2.33	0.00	0.47	99.31	0.401
*Tylocephalus*	0.21	0.14	1.45	3.33	1.00	0.44	99.75	0.635
*Clarkus*	0.12	0.18	0.67	1.67	0.00	0.25	100.00	0.92
*Ecphyadophora*	0.00	0.00	NaN	0.00	0.00	0.00	100.00	1
**R2M vs R2W**	**Average dissimilarity = 39.04**							
*Xenocriconemella*	7.54	5.70	1.32	16.00	92.00	19.32	19.32	0.016
Rhabditidae	6.56	2.40	2.73	93.67	30.00	16.80	36.12	0.261
*Meloidogyne*	4.42	2.85	1.55	76.00	33.67	11.32	47.44	0.228
*Gracilacus*	3.06	2.75	1.11	17.33	28.33	7.85	55.29	0.538
*Prismatolaimus*	2.67	1.86	1.44	20.67	41.67	6.85	62.14	0.161
*Acrobeloides*	2.52	2.16	1.17	36.33	37.67	6.46	68.60	0.209
*Filenchus*	1.66	0.80	2.07	133.00	123.33	4.25	72.85	0.949
*Alaimus*	1.54	1.27	1.21	20.00	16.00	3.93	76.78	0.45
*Ditylenchus*	1.30	0.89	1.45	15.33	13.33	3.33	80.11	0.245
*Acrobeles*	1.13	1.29	0.88	12.33	2.00	2.89	83.00	0.285
*Aphelenchoides*	0.96	0.68	1.42	18.00	16.67	2.46	85.46	0.936
Dorylaimida	0.94	0.74	1.28	4.33	10.67	2.42	87.88	0.103
*Boleodorus*	0.83	0.55	1.51	10.33	6.00	2.12	90.00	0.114
*Pseudacrobeles*	0.80	0.55	1.46	8.67	1.00	2.04	92.04	0.444
*Plectus*	0.69	0.35	1.95	10.67	17.67	1.77	93.81	0.979
*Aporcelaimellus*	0.44	0.39	1.13	3.67	2.00	1.12	94.93	0.171
*Clarkus*	0.44	0.39	1.10	4.00	0.00	1.11	96.04	0.026
*Ceratoplectus*	0.40	0.17	2.36	5.00	1.00	1.03	97.07	0.784
*Diphtherophora*	0.36	0.33	1.08	1.33	3.67	0.92	97.99	0.48
*Helicotylenchus*	0.24	0.28	0.87	1.00	2.00	0.62	98.61	0.921
*Cervidellus*	0.21	0.16	1.32	1.00	3.00	0.54	99.15	0.758
*Tylocephalus*	0.17	0.18	0.92	1.33	1.00	0.44	99.59	0.909
*Teratocephalus*	0.16	0.15	1.08	2.33	2.00	0.41	100.00	0.999
Diplogasteridae	0.00	0.00	NaN	0.00	0.00	0.00	100.00	1
*Ecphyadophora*	0.00	0.00	NaN	0.00	0.00	0.00	100.00	1
*Tripyla*	0.00	0.00	NaN	0.00	0.00	0.00	100.00	1
**Nov-17**								
**Control vs SLR**	**Average dissimilarity = 34.96**							
*Alaimus*	4.21	3.08	1.36	31.00	76.33	12.03	12.03	0.292
*Xenocriconemella*	4.16	3.50	1.19	95.00	112.00	11.91	23.94	0.922
*Meloidogyne*	3.55	1.46	2.42	47.33	58.67	10.15	34.09	0.514
*Helicotylenchus*	3.47	4.40	0.79	56.67	6.00	9.91	44.00	0.485
Rhabditidae	2.46	2.07	1.19	30.67	46.67	7.04	51.04	0.743
*Prismatolaimus*	2.21	0.85	2.61	43.67	37.33	6.32	57.36	0.587
*Gracilacus*	2.16	1.31	1.65	58.67	64.00	6.18	63.54	0.932
*Filenchus*	1.88	1.76	1.07	106.00	130.33	5.38	68.92	0.987
*Aphelenchoides*	1.38	1.21	1.14	17.00	27.00	3.94	72.86	0.41
*Ditylenchus*	1.31	0.60	2.20	21.33	4.33	3.76	76.62	0.521
*Acrobeles*	1.28	1.24	1.03	21.00	6.00	3.68	80.30	0.571
*Pseudacrobeles*	1.16	1.01	1.14	15.67	4.33	3.31	83.61	0.371
*Boleodorus*	0.93	0.62	1.51	4.00	14.00	2.67	86.28	0.258
*Acrobeloides*	0.69	0.62	1.12	14.67	11.67	1.98	88.26	0.86
*Plectus*	0.67	0.45	1.48	29.67	22.00	1.91	90.17	0.901
*Tripyla*	0.53	0.50	1.06	5.00	5.33	1.53	91.70	0.311
Dorylaimida	0.53	0.39	1.34	11.67	13.67	1.50	93.20	0.837
*Diphtherophora*	0.45	0.28	1.62	6.67	5.00	1.29	94.49	0.479
*Ceratoplectus*	0.42	0.28	1.52	8.33	8.33	1.20	95.69	0.778
*Aporcelaimellus*	0.37	0.37	1.00	4.00	7.33	1.05	96.74	0.652
*Teratocephalus*	0.30	0.33	0.90	3.33	2.67	0.86	97.60	0.218
*Clarkus*	0.28	0.28	1.01	3.33	6.67	0.80	98.40	0.903
*Tylocephalus*	0.17	0.14	1.27	2.33	1.33	0.50	98.90	0.737
*Ecphyadophora*	0.16	0.12	1.36	2.33	1.00	0.46	99.36	0.548
*Cervidellus*	0.14	0.15	0.96	1.67	1.00	0.41	99.77	0.744
Diplogasteridae	0.08	0.12	0.66	1.00	0.00	0.23	100.00	0.741
*Aphelenchus*	0.00	0.00	NaN	0.00	0.00	0.00	100.00	1
**Control vs R2M**	**Average dissimilarity = 43.16**							
*Xenocriconemella*	5.71	3.34	1.71	95.00	38.00	13.22	13.22	0.437
*Helicotylenchus*	4.38	4.92	0.89	56.67	1.00	10.14	23.36	0.008
*Meloidogyne*	4.06	3.20	1.27	47.33	51.67	9.39	32.75	0.289
*Filenchus*	3.60	2.07	1.74	106.00	142.67	8.35	41.10	0.705
Rhabditidae	3.42	2.80	1.22	30.67	60.67	7.93	49.03	0.476
*Alaimus*	3.29	2.70	1.22	31.00	44.33	7.62	56.65	0.66
*Gracilacus*	3.16	1.06	2.97	58.67	21.67	7.32	63.97	0.733
*Prismatolaimus*	2.88	1.91	1.51	43.67	24.67	6.67	70.64	0.133
*Acrobeles*	1.80	1.38	1.30	21.00	12.00	4.18	74.82	0.137
*Plectus*	1.53	0.52	2.96	29.67	11.67	3.54	78.36	0.023
*Pseudacrobeles*	1.32	1.07	1.23	15.67	10.33	3.05	81.41	0.193
*Acrobeloides*	1.15	0.77	1.49	14.67	15.00	2.66	84.07	0.381
*Ditylenchus*	0.98	0.79	1.24	21.33	10.67	2.27	86.34	0.691
Dorylaimida	0.87	0.58	1.50	11.67	2.67	2.01	88.35	0.08
*Aphelenchoides*	0.80	0.58	1.39	17.00	16.33	1.86	90.21	0.784
*Boleodorus*	0.73	0.36	2.03	4.00	11.33	1.70	91.91	0.72
*Ceratoplectus*	0.70	0.41	1.69	8.33	1.00	1.62	93.53	0.094
*Clarkus*	0.60	0.62	0.96	3.33	7.67	1.38	94.91	0.448
*Tripyla*	0.48	0.48	1.01	5.00	2.00	1.11	96.02	0.43
*Diphtherophora*	0.39	0.39	0.99	6.67	4.00	0.91	96.93	0.618
*Aporcelaimellus*	0.34	0.30	1.12	4.00	5.67	0.77	97.70	0.753
*Teratocephalus*	0.25	0.38	0.66	3.33	0.00	0.59	98.29	0.34
*Tylocephalus*	0.19	0.17	1.17	2.33	0.00	0.45	98.74	0.632
Diplogasteridae	0.19	0.22	0.89	1.00	1.67	0.45	99.19	0.27
*Ecphyadophora*	0.19	0.15	1.29	2.33	1.00	0.44	99.63	0.281
*Cervidellus*	0.16	0.17	0.94	1.67	1.00	0.37	100.00	0.674
*Aphelenchus*	0.00	0.00	NaN	0.00	0.00	0.00	100.00	1
**Control vs R2W**	**Average dissimilarity = 44.59**							
*Gracilacus*	7.54	4.60	1.64	58.67	72.00	16.90	16.90	0.188
*Xenocriconemella*	4.61	4.00	1.15	95.00	80.67	10.35	27.25	0.837
*Helicotylenchus*	4.37	5.12	0.85	56.67	3.00	9.80	37.05	0.006
*Filenchus*	4.35	2.55	1.71	106.00	63.67	9.75	46.80	0.432
*Meloidogyne*	4.19	2.23	1.88	47.33	55.00	9.40	56.20	0.222
*Prismatolaimus*	3.26	1.91	1.71	43.67	13.67	7.32	63.52	0.036
*Alaimus*	2.59	2.54	1.02	31.00	15.33	5.81	69.33	0.909
*Acrobeles*	2.02	1.46	1.39	21.00	1.00	4.53	73.86	0.07
Rhabditidae	1.92	1.66	1.16	30.67	20.67	4.31	78.17	0.838
*Plectus*	1.55	0.92	1.69	29.67	12.67	3.47	81.64	0.023
*Pseudacrobeles*	1.28	1.02	1.25	15.67	9.33	2.87	84.51	0.208
*Ditylenchus*	1.16	1.10	1.06	21.33	26.33	2.61	87.12	0.589
*Ceratoplectus*	0.82	0.40	2.08	8.33	0.00	1.84	88.96	0.024
Dorylaimida	0.68	0.50	1.35	11.67	5.67	1.52	90.48	0.491
*Acrobeloides*	0.59	0.41	1.45	14.67	18.00	1.33	91.81	0.932
*Aphelenchoides*	0.58	0.42	1.37	17.00	21.33	1.29	93.10	0.939
*Diphtherophora*	0.53	0.40	1.34	6.67	2.00	1.20	94.30	0.19
*Tripyla*	0.49	0.58	0.84	5.00	0.00	1.09	95.39	0.37
*Boleodorus*	0.46	0.57	0.80	4.00	1.00	1.03	96.42	0.981
*Clarkus*	0.32	0.23	1.37	3.33	3.67	0.71	97.13	0.853
*Cervidellus*	0.27	0.31	0.86	1.67	2.33	0.60	97.73	0.409
*Teratocephalus*	0.26	0.39	0.67	3.33	0.00	0.59	98.32	0.253
*Tylocephalus*	0.25	0.19	1.32	2.33	3.00	0.56	98.88	0.323
*Aporcelaimellus*	0.23	0.19	1.18	4.00	3.67	0.51	99.39	0.851
*Ecphyadophora*	0.18	0.13	1.39	2.33	1.67	0.39	99.78	0.408
Diplogasteridae	0.10	0.14	0.67	1.00	0.00	0.22	100.00	0.508
*Aphelenchus*	0.00	0.00	NaN	0.00	0.00	0.00	100.00	1
**SLR vs R2M**	**Average dissimilarity = 37.03**							
*Xenocriconemella*	6.87	3.94	1.75	112.00	38.00	18.55	18.55	0.12
*Alaimus*	3.85	3.53	1.09	76.33	44.33	10.41	28.96	0.458
Rhabditidae	3.48	3.06	1.14	46.67	60.67	9.39	38.35	0.429
*Gracilacus*	3.47	2.55	1.36	64.00	21.67	9.36	47.71	0.614
*Meloidogyne*	3.12	2.09	1.49	58.67	51.67	8.43	56.14	0.735
*Filenchus*	2.35	0.96	2.43	130.33	142.67	6.34	62.48	0.939
*Prismatolaimus*	1.81	0.96	1.89	37.33	24.67	4.87	67.35	0.894
*Aphelenchoides*	1.74	1.27	1.37	27.00	16.33	4.71	72.06	0.127
*Acrobeloides*	1.26	1.13	1.12	11.67	15.00	3.39	75.45	0.308
*Acrobeles*	1.15	1.08	1.06	6.00	12.00	3.10	78.55	0.719
Dorylaimida	0.92	0.52	1.76	13.67	2.67	2.48	81.03	0.06
*Boleodorus*	0.90	0.36	2.48	14.00	11.33	2.45	83.48	0.315
*Plectus*	0.84	0.45	1.89	22.00	11.67	2.28	85.76	0.695
*Ditylenchus*	0.84	0.63	1.33	4.33	10.67	2.26	88.02	0.829
*Pseudacrobeles*	0.67	0.66	1.01	4.33	10.33	1.81	89.83	0.81
*Clarkus*	0.67	0.49	1.38	6.67	7.67	1.80	91.63	0.336
*Ceratoplectus*	0.60	0.46	1.30	8.33	1.00	1.61	93.24	0.263
*Helicotylenchus*	0.50	0.45	1.12	6.00	1.00	1.36	94.60	0.91
*Tripyla*	0.48	0.53	0.90	5.33	2.00	1.28	95.88	0.44
*Aporcelaimellus*	0.47	0.46	1.01	7.33	5.67	1.26	97.14	0.376
*Diphtherophora*	0.35	0.21	1.67	5.00	4.00	0.95	98.09	0.728
*Teratocephalus*	0.20	0.31	0.66	2.67	0.00	0.55	98.64	0.612
Diplogasteridae	0.17	0.25	0.66	0.00	1.67	0.45	99.09	0.468
*Ecphyadophora*	0.12	0.15	0.82	1.00	1.00	0.33	99.42	0.881
*Cervidellus*	0.11	0.14	0.83	1.00	1.00	0.30	99.72	0.82
*Tylocephalus*	0.10	0.15	0.66	1.33	0.00	0.28	100.00	0.943
*Aphelenchus*	0.00	0.00	NaN	0.00	0.00	0.00	100.00	1
**SLR vs R2W**	**Average dissimilarity = 44.30**							
*Gracilacus*	7.45	4.77	1.56	64.00	72.00	16.81	16.81	0.207
*Filenchus*	6.06	2.35	2.58	130.33	63.67	13.67	30.48	0.045
*Alaimus*	5.75	3.18	1.81	76.33	15.33	12.97	43.45	0.035
*Xenocriconemella*	5.70	3.69	1.54	112.00	80.67	12.86	56.31	0.406
Rhabditidae	2.91	2.86	1.02	46.67	20.67	6.57	62.88	0.602
*Prismatolaimus*	2.15	0.96	2.25	37.33	13.67	4.86	67.74	0.621
*Ditylenchus*	2.03	1.49	1.36	4.33	26.33	4.57	72.31	0.05
*Meloidogyne*	1.93	1.42	1.36	58.67	55.00	4.36	76.67	0.99
*Aphelenchoides*	1.64	1.16	1.42	27.00	21.33	3.71	80.38	0.186
*Boleodorus*	1.16	0.82	1.42	14.00	1.00	2.61	82.99	0.033
*Acrobeloides*	0.90	0.85	1.06	11.67	18.00	2.04	85.03	0.593
*Plectus*	0.87	0.78	1.12	22.00	12.67	1.97	87.00	0.656
Dorylaimida	0.76	0.47	1.61	13.67	5.67	1.71	88.71	0.282
*Ceratoplectus*	0.71	0.44	1.62	8.33	0.00	1.60	90.31	0.071
*Pseudacrobeles*	0.60	0.46	1.31	4.33	9.33	1.36	91.67	0.87
*Helicotylenchus*	0.54	0.42	1.29	6.00	3.00	1.22	92.89	0.852
*Aporcelaimellus*	0.50	0.39	1.28	7.33	3.67	1.13	94.02	0.236
*Acrobeles*	0.49	0.33	1.46	6.00	1.00	1.10	95.12	0.932
*Clarkus*	0.46	0.28	1.67	6.67	3.67	1.04	96.16	0.59
*Tripyla*	0.43	0.64	0.67	5.33	0.00	0.96	97.12	0.591
*Diphtherophora*	0.42	0.34	1.24	5.00	2.00	0.95	98.07	0.56
*Tylocephalus*	0.26	0.22	1.14	1.33	3.00	0.58	98.65	0.306
*Cervidellus*	0.25	0.28	0.88	1.00	2.33	0.55	99.20	0.473
*Teratocephalus*	0.21	0.32	0.67	2.67	0.00	0.48	99.68	0.528
*Ecphyadophora*	0.14	0.12	1.17	1.00	1.67	0.32	100.00	0.753
*Aphelenchus*	0.00	0.00	NaN	0.00	0.00	0.00	100.00	1
Diplogasteridae	0.00	0.00	NaN	0.00	0.00	0.00	100.00	1
**R2M vs R2W**	**Average dissimilarity = 47.64**							
*Filenchus*	8.23	3.22	2.56	142.67	63.67	17.28	17.28	0.001
*Gracilacus*	7.35	8.75	0.84	21.67	72.00	15.43	32.71	0.227
*Xenocriconemella*	6.52	4.92	1.33	38.00	80.67	13.69	46.40	0.163
Rhabditidae	4.21	4.07	1.03	60.67	20.67	8.84	55.24	0.154
*Meloidogyne*	4.07	2.64	1.54	51.67	55.00	8.53	63.77	0.286
*Alaimus*	3.44	2.52	1.36	44.33	15.33	7.23	71.00	0.583
*Prismatolaimus*	1.87	1.58	1.18	24.67	13.67	3.91	74.91	0.816
*Ditylenchus*	1.79	1.69	1.06	10.67	26.33	3.77	78.68	0.183
*Acrobeloides*	1.45	0.84	1.72	15.00	18.00	3.04	81.72	0.111
*Acrobeles*	1.21	1.66	0.73	12.00	1.00	2.55	84.27	0.655
*Boleodorus*	1.10	0.34	3.27	11.33	1.00	2.30	86.57	0.068
*Aphelenchoides*	0.94	0.79	1.19	16.33	21.33	1.98	88.55	0.663
*Pseudacrobeles*	0.90	0.64	1.42	10.33	9.33	1.89	90.44	0.61
*Plectus*	0.80	0.59	1.37	11.67	12.67	1.69	92.13	0.755
*Clarkus*	0.80	0.72	1.11	7.67	3.67	1.68	93.81	0.102
*Aporcelaimellus*	0.49	0.32	1.54	5.67	3.67	1.03	94.84	0.278
Dorylaimida	0.42	0.29	1.44	2.67	5.67	0.88	95.72	0.955
*Diphtherophora*	0.38	0.20	1.86	4.00	2.00	0.80	96.52	0.653
*Helicotylenchus*	0.36	0.43	0.83	1.00	3.00	0.75	97.27	0.99
*Tylocephalus*	0.32	0.29	1.12	0.00	3.00	0.68	97.95	0.06
*Cervidellus*	0.29	0.34	0.85	1.00	2.33	0.61	98.56	0.219
Diplogasteridae	0.21	0.32	0.67	1.67	0.00	0.45	99.01	0.133
*Tripyla*	0.20	0.30	0.67	2.00	0.00	0.42	99.43	0.845
*Ecphyadophora*	0.17	0.15	1.15	1.00	1.67	0.36	99.79	0.478
*Ceratoplectus*	0.10	0.15	0.67	1.00	0.00	0.21	100.00	1
*Aphelenchus*	0.00	0.00	NaN	0.00	0.00	0.00	100.00	1
*Teratocephalus*	0.00	0.00	NaN	0.00	0.00	0.00	100.00	1
**Jan-18**								
**Control vs SLR**	**Average dissimilarity = 43.28**							
*Filenchus*	8.38	6.27	1.34	191.33	156.67	19.36	19.36	0.832
*Xenocriconemella*	6.54	3.87	1.69	57.67	108.67	15.11	34.47	0.807
*Gracilacus*	3.99	2.78	1.43	49.00	42.33	9.20	43.67	0.448
*Pseudacrobeles*	3.09	1.83	1.69	37.33	2.00	7.14	50.81	0.053
*Plectus*	2.33	2.17	1.07	40.67	21.00	5.37	56.18	0.532
*Ditylenchus*	2.05	2.06	1.00	28.00	9.00	4.74	60.92	0.317
*Prismatolaimus*	1.91	1.21	1.58	24.67	19.33	4.40	65.32	0.533
*Helicotylenchus*	1.88	1.46	1.29	23.33	2.33	4.35	69.67	0.087
*Acrobeloides*	1.86	1.60	1.16	23.33	22.00	4.30	73.97	0.71
*Aphelenchoides*	1.59	1.22	1.30	25.33	9.33	3.67	77.64	0.316
Rhabditidae	1.55	0.82	1.89	22.00	27.33	3.58	81.22	0.673
*Meloidogyne*	1.46	1.13	1.30	29.67	42.00	3.38	84.60	0.89
*Acrobeles*	1.37	1.71	0.80	2.67	16.67	3.17	87.77	0.49
*Tylocephalus*	0.83	0.76	1.09	9.00	2.33	1.91	89.68	0.249
*Ceratoplectus*	0.62	0.75	0.83	7.00	1.33	1.43	91.11	0.362
*Boleodorus*	0.62	0.58	1.07	6.33	0.00	1.43	92.54	0.089
*Ecphyadophora*	0.59	0.36	1.64	6.67	0.00	1.36	93.90	0.321
*Cervidellus*	0.48	0.37	1.30	5.67	1.33	1.12	95.02	0.257
*Teratocephalus*	0.47	0.43	1.09	5.33	2.00	1.07	96.09	0.4
*Alaimus*	0.43	0.22	1.93	10.00	8.00	1.00	97.09	0.916
*Aporcelaimellus*	0.34	0.30	1.17	4.00	2.33	0.79	97.88	0.603
*Clarkus*	0.26	0.39	0.66	3.33	0.00	0.60	98.48	0.509
*Diphtherophora*	0.24	0.28	0.86	2.33	1.00	0.56	99.04	0.409
Dorylaimida	0.22	0.33	0.65	2.33	0.00	0.50	99.54	0.422
Diplogasteridae	0.20	0.17	1.20	2.33	0.00	0.46	100.00	0.203
*Tripyla*	0.00	0.00	NaN	0.00	0.00	0.00	100.00	1
**Control vs R2M**	**Average dissimilarity = 49.59**							
*Filenchus*	10.97	4.62	2.38	191.33	93.33	22.12	22.12	0.239
*Xenocriconemella*	4.87	4.21	1.16	57.67	16.33	9.82	31.94	0.929
*Gracilacus*	4.61	4.77	0.97	49.00	11.00	9.29	41.23	0.299
*Plectus*	3.34	2.96	1.13	40.67	10.33	6.74	47.97	0.046
*Pseudacrobeles*	3.33	2.15	1.55	37.33	8.33	6.72	54.69	0.031
*Helicotylenchus*	2.40	1.85	1.30	23.33	0.67	4.84	59.53	0.018
*Meloidogyne*	2.39	1.34	1.78	29.67	10.33	4.82	64.35	0.429
*Prismatolaimus*	2.37	2.02	1.17	24.67	6.00	4.78	69.13	0.144
*Acrobeloides*	2.13	1.97	1.08	23.33	18.67	4.29	73.42	0.544
*Ditylenchus*	2.12	2.23	0.95	28.00	19.67	4.28	77.70	0.314
*Aphelenchoides*	2.06	1.67	1.24	25.33	9.67	4.16	81.86	0.097
*Alaimus*	1.19	0.54	2.19	10.00	0.67	2.41	84.27	0.056
*Tylocephalus*	1.18	1.15	1.03	9.00	0.00	2.38	86.65	0.013
Rhabditidae	0.98	0.82	1.20	22.00	15.33	1.99	88.64	0.956
*Boleodorus*	0.81	0.72	1.11	6.33	0.00	1.62	90.26	0.01
*Ceratoplectus*	0.81	1.01	0.80	7.00	0.00	1.63	91.89	0.034
*Acrobeles*	0.76	0.67	1.13	2.67	5.67	1.54	93.43	0.736
*Cervidellus*	0.59	0.38	1.57	5.67	2.00	1.19	94.62	0.068
*Ecphyadophora*	0.58	0.45	1.30	6.67	3.33	1.17	95.79	0.373
*Teratocephalus*	0.51	0.58	0.89	5.33	0.67	1.03	96.82	0.22
*Clarkus*	0.39	0.35	1.13	3.33	1.33	0.80	97.62	0.086
*Aporcelaimellus*	0.39	0.25	1.57	4.00	2.00	0.78	98.40	0.445
*Diphtherophora*	0.28	0.42	0.66	2.33	0.00	0.56	98.96	0.282
Dorylaimida	0.28	0.42	0.66	2.33	0.00	0.56	99.52	0.025
Diplogasteridae	0.24	0.19	1.28	2.33	0.67	0.48	100.00	0.062
*Tripyla*	0.00	0.00	NaN	0.00	0.00	0.00	100.00	1
**Control vs R2W**	**Average dissimilarity = 49.09**							
*Filenchus*	10.40	5.81	1.79	191.33	88.33	21.18	21.18	0.359
*Xenocriconemella*	8.59	9.06	0.95	57.67	93.67	17.50	38.68	0.605
*Gracilacus*	4.08	4.45	0.92	49.00	14.67	8.31	46.99	0.437
*Plectus*	3.33	2.86	1.17	40.67	5.67	6.78	53.77	0.062
*Pseudacrobeles*	2.94	1.88	1.56	37.33	12.33	5.99	59.76	0.074
*Prismatolaimus*	2.39	2.22	1.08	24.67	1.00	4.87	64.63	0.154
*Helicotylenchus*	2.22	1.67	1.33	23.33	1.00	4.53	69.16	0.03
*Acrobeloides*	2.22	1.54	1.45	23.33	18.33	4.53	73.69	0.441
*Ditylenchus*	2.13	2.09	1.02	28.00	13.67	4.34	78.03	0.263
*Meloidogyne*	1.57	1.04	1.50	29.67	16.33	3.19	81.22	0.825
*Aphelenchoides*	1.29	0.89	1.44	25.33	16.33	2.63	83.85	0.497
*Tylocephalus*	1.05	0.97	1.08	9.00	0.67	2.14	85.99	0.034
Rhabditidae	1.03	0.77	1.34	22.00	23.33	2.09	88.08	0.945
*Alaimus*	0.80	0.41	1.95	10.00	3.00	1.63	89.71	0.409
*Ceratoplectus*	0.74	0.93	0.80	7.00	0.00	1.51	91.22	0.082
*Boleodorus*	0.74	0.67	1.10	6.33	0.00	1.51	92.73	0.026
*Ecphyadophora*	0.70	0.41	1.70	6.67	0.00	1.42	94.15	0.142
*Cervidellus*	0.57	0.38	1.53	5.67	1.00	1.17	95.32	0.077
*Teratocephalus*	0.48	0.54	0.88	5.33	0.67	0.98	96.30	0.325
*Aporcelaimellus*	0.44	0.33	1.31	4.00	0.00	0.89	97.19	0.234
*Acrobeles*	0.32	0.30	1.05	2.67	0.00	0.65	97.84	0.788
*Clarkus*	0.30	0.45	0.66	3.33	0.00	0.60	98.44	0.356
*Diphtherophora*	0.28	0.34	0.81	2.33	0.67	0.56	99.00	0.268
Dorylaimida	0.25	0.38	0.66	2.33	0.00	0.52	99.52	0.126
Diplogasteridae	0.23	0.19	1.22	2.33	0.00	0.48	100.00	0.063
*Tripyla*	0.00	0.00	NaN	0.00	0.00	0.00	100.00	1
**SLR vs R2M**	**Average dissimilarity = 52.55**							
*Xenocriconemella*	12.82	5.49	2.34	108.67	16.33	24.39	24.39	0.183
*Filenchus*	12.77	4.88	2.62	156.67	93.33	24.30	48.69	0.068
*Meloidogyne*	4.90	2.73	1.79	42.00	10.33	9.33	58.02	0.002
*Gracilacus*	4.70	2.43	1.93	42.33	11.00	8.93	66.95	0.254
Rhabditidae	2.48	1.50	1.66	27.33	15.33	4.72	71.67	0.138
*Acrobeles*	2.21	2.29	0.96	16.67	5.67	4.20	75.87	0.081
*Acrobeloides*	2.08	2.38	0.87	22.00	18.67	3.95	79.82	0.607
*Plectus*	1.69	1.16	1.47	21.00	10.33	3.23	83.05	0.602
*Prismatolaimus*	1.66	0.92	1.81	19.33	6.00	3.16	86.21	0.567
*Ditylenchus*	1.63	1.24	1.31	9.00	19.67	3.09	89.30	0.53
*Alaimus*	1.24	1.12	1.11	8.00	0.67	2.36	91.66	0.034
*Pseudacrobeles*	0.96	0.74	1.29	2.00	8.33	1.82	93.48	0.94
*Aphelenchoides*	0.79	0.61	1.28	9.33	9.67	1.50	94.98	0.909
*Ecphyadophora*	0.52	0.63	0.81	0.00	3.33	0.98	95.96	0.488
*Aporcelaimellus*	0.41	0.32	1.29	2.33	2.00	0.78	96.74	0.361
*Helicotylenchus*	0.30	0.35	0.87	2.33	0.67	0.58	97.32	0.883
*Teratocephalus*	0.30	0.32	0.94	2.00	0.67	0.57	97.89	0.657
*Tylocephalus*	0.28	0.21	1.31	2.33	0.00	0.53	98.42	0.766
*Cervidellus*	0.28	0.24	1.14	1.33	2.00	0.53	98.95	0.885
*Clarkus*	0.19	0.16	1.19	0.00	1.33	0.36	99.31	0.563
*Ceratoplectus*	0.15	0.23	0.66	1.33	0.00	0.29	99.60	0.64
*Diphtherophora*	0.13	0.19	0.66	1.00	0.00	0.24	99.84	0.631
Diplogasteridae	0.08	0.13	0.64	0.00	0.67	0.16	100.00	0.856
*Boleodorus*	0.00	0.00	NaN	0.00	0.00	0.00	100.00	1
Dorylaimida	0.00	0.00	NaN	0.00	0.00	0.00	100.00	1
*Tripyla*	0.00	0.00	NaN	0.00	0.00	0.00	100.00	1
**SLR vs R2W**	**Average dissimilarity = 47.81**							
*Xenocriconemella*	12.65	5.45	2.32	108.67	93.67	26.46	26.46	0.22
*Filenchus*	11.72	6.94	1.69	156.67	88.33	24.51	50.97	0.181
*Gracilacus*	4.31	2.28	1.90	42.33	14.67	9.02	59.99	0.352
*Meloidogyne*	3.53	2.10	1.69	42.00	16.33	7.40	67.39	0.053
Rhabditidae	2.28	1.69	1.35	27.33	23.33	4.76	72.15	0.196
*Prismatolaimus*	2.18	0.73	2.98	19.33	1.00	4.57	76.72	0.33
*Acrobeloides*	2.10	1.77	1.18	22.00	18.33	4.38	81.10	0.54
*Plectus*	1.84	0.93	1.98	21.00	5.67	3.85	84.95	0.54
*Acrobeles*	1.79	2.45	0.73	16.67	0.00	3.76	88.71	0.309
*Pseudacrobeles*	1.29	1.13	1.14	2.00	12.33	2.70	91.41	0.853
*Aphelenchoides*	1.09	1.00	1.09	9.33	16.33	2.27	93.68	0.681
*Alaimus*	0.78	0.83	0.94	8.00	3.00	1.63	95.31	0.448
*Ditylenchus*	0.68	0.57	1.20	9.00	13.67	1.41	96.72	0.951
*Helicotylenchus*	0.31	0.32	0.99	2.33	1.00	0.66	97.38	0.849
*Teratocephalus*	0.27	0.29	0.91	2.00	0.67	0.56	97.94	0.711
*Tylocephalus*	0.24	0.17	1.44	2.33	0.67	0.51	98.45	0.814
*Aporcelaimellus*	0.24	0.37	0.66	2.33	0.00	0.52	98.97	0.893
*Cervidellus*	0.21	0.23	0.90	1.33	1.00	0.43	99.40	0.952
*Diphtherophora*	0.15	0.16	0.90	1.00	0.67	0.31	99.71	0.566
*Ceratoplectus*	0.14	0.21	0.66	1.33	0.00	0.29	100.00	0.696
*Boleodorus*	0.00	0.00	NaN	0.00	0.00	0.00	100.00	1
*Clarkus*	0.00	0.00	NaN	0.00	0.00	0.00	100.00	1
Diplogasteridae	0.00	0.00	NaN	0.00	0.00	0.00	100.00	1
Dorylaimida	0.00	0.00	NaN	0.00	0.00	0.00	100.00	1
*Ecphyadophora*	0.00	0.00	NaN	0.00	0.00	0.00	100.00	1
*Tripyla*	0.00	0.00	NaN	0.00	0.00	0.00	100.00	1
**R2M vs R2W**	**Average dissimilarity = 39.42**							
*Xenocriconemella*	13.46	15.76	0.85	16.33	93.67	34.14	34.14	0.148
*Filenchus*	7.11	4.32	1.65	93.33	88.33	18.03	52.17	0.946
*Rhabditidae*	2.41	1.95	1.24	15.33	23.33	6.12	58.29	0.155
*Acrobeloides*	2.33	1.30	1.80	18.67	18.33	5.92	64.21	0.392
*Gracilacus*	2.02	1.05	1.93	11.00	14.67	5.12	69.33	0.931
*Ditylenchus*	1.79	0.65	2.77	19.67	13.67	4.54	73.87	0.484
*Aphelenchoides*	1.64	1.53	1.07	9.67	16.33	4.17	78.04	0.281
*Meloidogyne*	1.43	0.97	1.48	10.33	16.33	3.64	81.68	0.92
*Pseudacrobeles*	1.37	1.22	1.13	8.33	12.33	3.47	85.15	0.83
*Plectus*	0.98	0.89	1.10	10.33	5.67	2.49	87.64	0.961
*Prismatolaimus*	0.96	0.50	1.90	6.00	1.00	2.43	90.07	0.934
*Acrobeles*	0.91	1.37	0.66	5.67	0.00	2.30	92.37	0.647
*Ecphyadophora*	0.68	0.81	0.84	3.33	0.00	1.73	94.10	0.182
*Alaimus*	0.58	0.46	1.25	0.67	3.00	1.47	95.57	0.782
*Aporcelaimellus*	0.39	0.39	1.02	2.00	0.00	1.00	96.57	0.411
*Cervidellus*	0.34	0.27	1.26	2.00	1.00	0.86	97.43	0.771
*Clarkus*	0.25	0.20	1.25	1.33	0.00	0.64	98.07	0.472
*Helicotylenchus*	0.24	0.29	0.83	0.67	1.00	0.62	98.69	0.908
*Teratocephalus*	0.18	0.21	0.82	0.67	0.67	0.44	99.13	0.832
*Tylocephalus*	0.13	0.20	0.66	0.00	0.67	0.34	99.47	0.935
Diplogasteridae	0.11	0.16	0.66	0.67	0.00	0.27	99.74	0.686
*Diphtherophora*	0.10	0.15	0.66	0.00	0.67	0.26	100.00	0.768
*Boleodorus*	0.00	0.00	NaN	0.00	0.00	0.00	100.00	1
*Ceratoplectus*	0.00	0.00	NaN	0.00	0.00	0.00	100.00	1
Dorylaimida	0.00	0.00	NaN	0.00	0.00	0.00	100.00	1
*Tripyla*	0.00	0.00	NaN	0.00	0.00	0.00	100.00	1
**May-18**								
**Control vs SLR**	**Average dissimilarity = 40.20**							
*Meloidogyne*	7.31	5.99	1.22	135.33	140.67	18.17	18.17	0.85
*Filenchus*	4.87	3.14	1.55	130.67	76.67	12.12	30.29	0.822
*Xenocriconemella*	3.72	3.58	1.04	18.00	46.67	9.24	39.53	0.749
*Acrobeles*	2.66	1.74	1.53	32.67	9.67	6.60	46.13	0.116
*Gracilacus*	2.10	0.94	2.24	28.33	4.33	5.22	51.35	0.057
*Helicotylenchus*	2.03	2.48	0.82	22.33	3.67	5.05	56.40	0.414
*Rhabditidae*	2.02	1.56	1.30	46.00	34.00	5.02	61.42	0.525
*Plectus*	2.01	1.40	1.44	45.67	24.67	4.99	66.41	0.669
*Prismatolaimus*	1.78	1.53	1.17	21.67	30.67	4.43	70.84	0.538
*Ditylenchus*	1.76	1.30	1.35	24.33	4.33	4.37	75.21	0.054
*Alaimus*	1.69	1.07	1.59	24.33	38.00	4.20	79.41	0.871
*Ceratoplectus*	1.08	0.63	1.72	15.00	3.33	2.68	82.09	0.127
*Acrobeloides*	0.98	0.70	1.40	22.33	18.00	2.44	84.53	0.96
*Pseudacrobeles*	0.96	0.43	2.23	14.67	4.00	2.38	86.91	0.09
*Aphelenchoides*	0.92	0.63	1.47	15.00	5.67	2.29	89.20	0.822
*Boleodorus*	0.90	0.52	1.73	12.67	3.00	2.22	91.42	0.091
*Aporcelaimellus*	0.51	0.35	1.44	6.00	1.67	1.26	92.68	0.141
*Tripyla*	0.50	0.44	1.15	5.00	2.33	1.26	93.94	0.261
Dorylaimida	0.47	0.35	1.34	8.67	7.00	1.15	95.09	0.79
*Clarkus*	0.46	0.31	1.45	5.67	1.67	1.14	96.23	0.12
*Cervidellus*	0.44	0.31	1.42	5.00	0.67	1.09	97.32	0.37
*Diphtherophora*	0.37	0.29	1.28	2.67	4.33	0.93	98.25	0.595
*Tylocephalus*	0.27	0.22	1.23	3.33	0.67	0.68	98.93	0.262
Diplogasteridae	0.20	0.29	0.66	2.33	0.00	0.49	99.42	0.473
*Ecphyadophora*	0.12	0.18	0.66	1.33	0.00	0.29	99.71	0.83
*Teratocephalus*	0.12	0.18	0.66	1.33	0.00	0.29	100.00	0.735
**Control vs R2M**	**Average dissimilarity = 42.25**							
*Meloidogyne*	7.81	5.32	1.47	135.33	190.67	18.47	18.47	0.762
*Filenchus*	7.76	2.49	3.12	130.67	49.33	18.37	36.84	0.083
*Acrobeles*	2.93	2.17	1.35	32.67	5.67	6.94	43.78	0.052
*Gracilacus*	2.17	1.19	1.82	28.33	4.67	5.13	48.91	0.041
*Helicotylenchus*	2.07	2.89	0.72	22.33	0.00	4.91	53.82	0.374
*Alaimus*	1.84	1.47	1.25	24.33	6.67	4.36	58.18	0.828
*Plectus*	1.69	1.40	1.21	45.67	28.00	4.00	62.18	0.845
*Acrobeloides*	1.63	0.81	2.01	22.33	32.67	3.86	66.04	0.609
*Xenocriconemella*	1.49	1.50	0.99	18.00	10.00	3.52	69.56	0.887
*Ceratoplectus*	1.38	0.70	1.96	15.00	1.00	3.27	72.83	0.012
*Rhabditidae*	1.37	1.48	0.92	46.00	33.33	3.25	76.08	0.855
*Ditylenchus*	1.35	1.29	1.05	24.33	9.67	3.19	79.27	0.433
*Prismatolaimus*	1.31	1.19	1.11	21.67	10.00	3.11	82.38	0.771
*Boleodorus*	1.18	0.34	3.48	12.67	0.00	2.79	85.17	0.011
*Aphelenchoides*	1.16	0.94	1.24	15.00	23.33	2.75	87.92	0.749
*Pseudacrobeles*	1.13	0.31	3.68	14.67	2.67	2.67	90.59	0.011
Dorylaimida	0.79	0.44	1.80	8.67	0.00	1.87	92.46	0.093
*Aporcelaimellus*	0.59	0.51	1.16	6.00	0.00	1.41	93.87	0.042
*Clarkus*	0.53	0.43	1.24	5.67	0.00	1.26	95.13	0.036
*Cervidellus*	0.48	0.38	1.28	5.00	3.33	1.14	96.27	0.227
*Tripyla*	0.44	0.66	0.66	5.00	0.00	1.04	97.31	0.429
*Diphtherophora*	0.30	0.37	0.80	2.67	0.67	0.70	98.01	0.841
*Tylocephalus*	0.28	0.25	1.11	3.33	1.00	0.67	98.68	0.195
*Ecphyadophora*	0.21	0.24	0.88	1.33	1.67	0.49	99.17	0.534
Diplogasteridae	0.20	0.31	0.66	2.33	0.00	0.48	99.65	0.329
*Teratocephalus*	0.15	0.15	0.94	1.33	0.67	0.35	100.00	0.621
**Control vs R2W**	**Average dissimilarity = 56.30**							
*Filenchus*	11.48	1.83	6.26	130.67	25.00	20.38	20.38	0.001
*Meloidogyne*	7.47	4.99	1.50	135.33	114.33	13.27	33.65	0.837
*Xenocriconemella*	4.27	3.64	1.17	18.00	47.33	7.59	41.24	0.663
*Plectus*	4.23	1.51	2.80	45.67	6.67	7.51	48.75	0.002
*Acrobeles*	3.57	2.73	1.31	32.67	0.00	6.35	55.10	0.013
*Alaimus*	2.77	1.63	1.70	24.33	0.00	4.91	60.01	0.239
*Rhabditidae*	2.62	1.77	1.48	46.00	21.00	4.65	64.66	0.1
*Gracilacus*	2.59	1.22	2.12	28.33	3.67	4.60	69.26	0.003
*Helicotylenchus*	2.36	3.04	0.78	22.33	3.67	4.19	73.45	0.124
*Prismatolaimus*	2.06	1.44	1.43	21.67	1.67	3.67	77.12	0.482
*Ceratoplectus*	1.70	0.79	2.14	15.00	0.00	3.01	80.13	0.001
*Ditylenchus*	1.64	1.49	1.10	24.33	8.67	2.92	83.05	0.12
*Boleodorus*	1.35	0.36	3.72	12.67	0.00	2.40	85.45	0.001
*Pseudacrobeles*	1.28	0.40	3.19	14.67	3.00	2.27	87.72	0.003
*Acrobeloides*	1.08	0.77	1.40	22.33	14.00	1.92	89.64	0.927
*Aphelenchoides*	1.02	0.81	1.26	15.00	16.67	1.81	91.45	0.813
Dorylaimida	0.90	0.49	1.84	8.67	0.00	1.60	93.05	0.032
*Aporcelaimellus*	0.69	0.60	1.15	6.00	0.00	1.23	94.28	0.009
*Clarkus*	0.61	0.49	1.26	5.67	0.00	1.08	95.36	0.009
*Cervidellus*	0.56	0.44	1.27	5.00	0.00	1.01	96.37	0.116
*Tripyla*	0.50	0.75	0.66	5.00	0.00	0.88	97.25	0.27
*Diphtherophora*	0.40	0.32	1.24	2.67	2.33	0.70	97.95	0.551
*Tylocephalus*	0.36	0.30	1.17	3.33	0.00	0.64	98.59	0.021
*Teratocephalus*	0.35	0.39	0.89	1.33	2.67	0.62	99.21	0.436
Diplogasteridae	0.27	0.30	0.90	2.33	1.00	0.48	99.69	0.131
*Ecphyadophora*	0.18	0.20	0.90	1.33	1.00	0.31	100.00	0.573
**SLR vs R2M**	**Average dissimilarity = 38.70**							
*Meloidogyne*	9.87	6.15	1.60	140.67	190.67	25.52	25.52	0.292
*Xenocriconemella*	4.48	5.67	0.79	46.67	10.00	11.58	37.10	0.566
*Filenchus*	4.14	4.30	0.96	76.67	49.33	10.69	47.79	0.924
*Alaimus*	3.62	1.96	1.85	38.00	6.67	9.35	57.14	0.055
*Prismatolaimus*	2.29	2.25	1.02	30.67	10.00	5.93	63.07	0.384
*Acrobeloides*	2.25	1.49	1.51	18.00	32.67	5.82	68.89	0.16
Rhabditidae	1.88	1.17	1.62	34.00	33.33	4.87	73.76	0.59
*Aphelenchoides*	1.88	1.38	1.36	5.67	23.33	4.86	78.62	0.143
*Plectus*	1.32	0.70	1.89	24.67	28.00	3.42	82.04	0.96
*Acrobeles*	1.06	0.86	1.24	9.67	5.67	2.75	84.79	0.825
*Ditylenchus*	0.86	0.55	1.58	4.33	9.67	2.23	87.02	0.631
Dorylaimida	0.78	0.42	1.87	7.00	0.00	2.03	89.05	0.119
*Gracilacus*	0.72	0.36	1.98	4.33	4.67	1.85	90.90	0.969
*Diphtherophora*	0.50	0.35	1.42	4.33	0.67	1.29	92.19	0.158
*Helicotylenchus*	0.45	0.68	0.66	3.67	0.00	1.16	93.35	0.819
*Pseudacrobeles*	0.43	0.36	1.18	4.00	2.67	1.11	94.46	0.97
*Ceratoplectus*	0.37	0.36	1.03	3.33	1.00	0.95	95.41	0.908
*Cervidellus*	0.37	0.43	0.84	0.67	3.33	0.95	96.36	0.551
*Boleodorus*	0.30	0.46	0.66	3.00	0.00	0.78	97.14	0.918
*Tripyla*	0.29	0.23	1.27	2.33	0.00	0.74	97.88	0.538
*Ecphyadophora*	0.22	0.33	0.66	0.00	1.67	0.56	98.44	0.527
*Aporcelaimellus*	0.21	0.17	1.23	1.67	0.00	0.53	98.97	0.744
*Clarkus*	0.18	0.14	1.29	1.67	0.00	0.48	99.45	0.79
*Tylocephalus*	0.13	0.14	0.96	0.67	1.00	0.35	99.80	0.755
*Teratocephalus*	0.08	0.12	0.66	0.00	0.67	0.20	100.00	0.869
Diplogasteridae	0.00	0.00	NaN	0.00	0.00	0.00	100.00	1
**SLR vs R2W**	**Average dissimilarity = 48.26**							
*Meloidogyne*	9.87	6.17	1.60	140.67	114.33	20.44	20.44	0.322
*Filenchus*	7.28	5.61	1.30	76.67	25.00	15.09	35.53	0.142
*Xenocriconemella*	6.11	6.25	0.98	46.67	47.33	12.67	48.20	0.124
*Alaimus*	5.12	1.94	2.64	38.00	0.00	10.62	58.82	0.002
*Prismatolaimus*	3.69	2.43	1.52	30.67	1.67	7.65	66.47	0.005
Rhabditidae	2.54	1.42	1.79	34.00	21.00	5.27	71.74	0.141
*Plectus*	2.51	1.93	1.30	24.67	6.67	5.19	76.93	0.339
*Aphelenchoides*	1.63	1.40	1.17	5.67	16.67	3.38	80.31	0.356
*Acrobeloides*	1.47	1.10	1.33	18.00	14.00	3.05	83.36	0.726
*Acrobeles*	1.23	1.03	1.19	9.67	0.00	2.54	85.90	0.793
*Ditylenchus*	0.98	0.49	2.02	4.33	8.67	2.03	87.93	0.55
Dorylaimida	0.93	0.49	1.91	7.00	0.00	1.92	89.85	0.03
*Gracilacus*	0.72	0.28	2.59	4.33	3.67	1.49	91.34	0.973
*Helicotylenchus*	0.69	0.59	1.16	3.67	3.67	1.42	92.76	0.575
*Pseudacrobeles*	0.52	0.42	1.24	4.00	3.00	1.08	93.84	0.894
*Diphtherophora*	0.49	0.32	1.54	4.33	2.33	1.02	94.86	0.186
*Ceratoplectus*	0.49	0.45	1.09	3.33	0.00	1.02	95.88	0.79
*Teratocephalus*	0.39	0.59	0.66	0.00	2.67	0.80	96.68	0.399
*Boleodorus*	0.35	0.53	0.66	3.00	0.00	0.74	97.42	0.855
*Tripyla*	0.35	0.27	1.28	2.33	0.00	0.71	98.13	0.501
*Aporcelaimellus*	0.25	0.20	1.24	1.67	0.00	0.51	98.64	0.665
*Clarkus*	0.22	0.17	1.30	1.67	0.00	0.45	99.09	0.75
Diplogasteridae	0.12	0.18	0.66	0.00	1.00	0.25	99.34	0.714
*Ecphyadophora*	0.12	0.18	0.66	0.00	1.00	0.25	99.59	0.776
*Cervidellus*	0.10	0.15	0.66	0.67	0.00	0.21	99.80	0.915
*Tylocephalus*	0.10	0.15	0.66	0.67	0.00	0.20	100.00	0.906
**R2M vs R2W**	**Average dissimilarity = 37.48**							
*Meloidogyne*	11.64	7.20	1.62	190.67	114.33	31.06	31.06	0.099
*Xenocriconemella*	5.22	5.87	0.89	10.00	47.33	13.93	44.99	0.323
*Filenchus*	3.88	2.25	1.72	49.33	25.00	10.35	55.34	0.941
*Plectus*	3.08	0.83	3.69	28.00	6.67	8.22	63.56	0.093
*Acrobeloides*	2.99	1.58	1.89	32.67	14.00	7.96	71.52	0.014
*Aphelenchoides*	1.90	1.40	1.36	23.33	16.67	5.08	76.60	0.135
Rhabditidae	1.73	0.85	2.04	33.33	21.00	4.61	81.21	0.703
*Prismatolaimus*	1.27	0.88	1.44	10.00	1.67	3.39	84.60	0.815
*Gracilacus*	0.90	0.99	0.91	4.67	3.67	2.40	87.00	0.873
*Alaimus*	0.88	0.75	1.18	6.67	0.00	2.35	89.35	0.997
*Acrobeles*	0.69	1.04	0.66	5.67	0.00	1.85	91.20	0.943
*Helicotylenchus*	0.55	0.58	0.96	0.00	3.67	1.48	92.68	0.663
*Ditylenchus*	0.51	0.45	1.14	9.67	8.67	1.35	94.03	0.971
*Teratocephalus*	0.45	0.56	0.81	0.67	2.67	1.21	95.24	0.189
*Cervidellus*	0.41	0.61	0.66	3.33	0.00	1.09	96.33	0.453
*Pseudacrobeles*	0.35	0.33	1.07	2.67	3.00	0.94	97.27	0.987
*Diphtherophora*	0.33	0.32	1.03	0.67	2.33	0.88	98.15	0.761
*Ecphyadophora*	0.32	0.38	0.84	1.67	1.00	0.85	99.00	0.118
Diplogasteridae	0.13	0.20	0.66	0.00	1.00	0.35	99.35	0.6
*Ceratoplectus*	0.12	0.18	0.66	1.00	0.00	0.32	99.67	0.997
*Tylocephalus*	0.12	0.18	0.66	1.00	0.00	0.33	100.00	0.781
*Aporcelaimellus*	0.00	0.00	NaN	0.00	0.00	0.00	100.00	1
*Boleodorus*	0.00	0.00	NaN	0.00	0.00	0.00	100.00	1
*Clarkus*	0.00	0.00	NaN	0.00	0.00	0.00	100.00	1
Dorylaimida	0.00	0.00	NaN	0.00	0.00	0.00	100.00	1
*Tripyla*	0.00	0.00	NaN	0.00	0.00	0.00	100.00	1
